# *Momordica charantia* L. (Cucurbitaceae) Leaf Extract from Phytochemical Characterization and Toxicity Evaluation to Modulation of Pro-Inflammatory Cytokines and MAPK/NFκB Pathways

**DOI:** 10.3390/molecules30224335

**Published:** 2025-11-07

**Authors:** Maria Lúcia de Azevedo Oliveira, Rubiamara Mauricio de Sousa, Eder Alves Barbosa, Ony Araújo Galdino, Duanny Lorena Aires Dantas, Ingrid Reale Alves, Raphaelle Sousa Borges, Nayara Costa de Melo Castelo Branco, Artemis Socorro do Nascimento Rodrigues, Gisele Custódio de Souza, Saulo Victor e Silva, Gabriel Araujo-Silva, Jefferson Romáryo Duarte da Luz, Maria das Graças Almeida

**Affiliations:** 1 Post-Graduation Program in Pharmaceutical Sciences, Health Sciences Center, Federal University of Rio Grande do Norte, R. Gen. Gustavo Cordeiro de Farias, s/n—Petrópolis, Natal 59012-570, RN, Brazil; maluciaaazevedo@gmail.com (M.L.d.A.O.); mgalmeida84@gmail.com (M.d.G.A.); 2 Multidisciplinary Research Laboratory, Department of Clinical and Toxicological Analysis, Health Sciences Center, Federal University of Rio Grande do Norte, R. Gen. Gustavo Cordeiro de Farias, s/n—Petrópolis, Natal 59012-570, RN, Brazil; rubiamaramds1@gmail.com (R.M.d.S.);; 3 Laboratory of Synthesis and Analysis of Biomolecules (LSAB), Institute of Chemistry, Darcy Ribeiro University Campus, University of Brasilia, Brasília 70910-900, DF, Brazil; 4 Department of Biological and Health Sciences—DCBS, Federal University of Amapá (UNIFAP), Rodovia Rod. Josmar Chaves Pinto, km 02—Jardim Marco Zero, Macapá 68903-419, AP, Brazil; ingridreale10@gmail.com (I.R.A.); raphaellebio@yahoo.com.br (R.S.B.); nayara@unifap.br (N.C.d.M.C.B.); artemis@unifap.br (A.S.d.N.R.); 5 College of Natural Sciences, Amapá State University (UEAP), Av. Presidente Vargas, s/n—Centro, Macapá 68900-070, AP, Brazil; gisele.souza@ueap.edu.br; 6 Department of Food Science and Nutrition, University of Antofagasta, Antofagasta 02800, Chile; saulo.silva@uantof.cl; 7 Organic Chemistry and Biochemistry Laboratory, Amapá State University (UEAP), Av. Presidente Vargas, s/n—Centro, Macapá 68900-070, AP, Brazil; gabriel.silva@ueap.edu.br; 8 College of Biological Sciences, Federal University of Amapá (UNIFAP), Rodovia BR 156, Universidade, Oiapoque 68980-000, AP, Brazil

**Keywords:** *Momordica charantia*, cytokines, nitric oxide, acute toxicity, peritonitis model, RAW 264.7 macrophages, anti-inflammatory activity

## Abstract

*Momordica charantia* L. (Cucurbitaceae) has been widely recognized for its pharmacological potential, although studies on its leaves remain scarce. In this study, the hydroethanolic leaf extract (MCHLE) was chemically characterized by LC–MS/MS, revealing the presence of octopamine, ferulate, vitexin-2-O-rhamnoside, and other bioactive phenolics. Toxicological evaluation in *Wistar* rats demonstrated that both acute (2000 mg/kg) and repeated oral administration (up to 400 mg/kg for 28 days) caused no clinical or behavioral signs of toxicity. Notably, treatment significantly reduced glucose and cholesterol levels, in addition to attenuating lipid peroxidation and enhancing antioxidant defenses. *In vivo*, MCHLE inhibited leukocyte and neutrophil infiltration in the LPS-induced peritonitis model, with efficacy comparable to dexamethasone. It also reduced TNF-α secretion and nitric oxide generation in peritoneal fluids. In vitro assays with LPS-stimulated RAW 264.7 macrophages confirmed these effects, showing dose-dependent inhibition of TNF-α, IL-1β, and NO production. Gene expression analysis further demonstrated downregulation of TNF-α and MAPK, with marked suppression of NF-κB transcripts. Collectively, these results suggest that MCHLE exerts anti-inflammatory activity by targeting both mediator release and upstream signaling pathways, while maintaining a favorable safety profile, supporting its potential for further investigation as a promising source of bioactive compounds.

## 1. Introduction

Inflammation is a physiological defense process that protects the organism from infection and tissue injury. Its purpose is to eliminate harmful stimuli, promote repair and healing, and establish immune memory when infection occurs. This response is normally beneficial to the body, being self-regulated, restoring homeostasis through the interaction between immune and structural cells and the production of chemical mediators that neutralize stimuli, such as microbial products (lipopolysaccharides—LPS, lipoteichoic acid, flagellin) or endogenous molecules that affect tissue homeostasis [1].

In the acute phase, inflammation, which begins immediately after the injury, increases blood flow and vascular permeability, with leukocyte recruitment at the site of the injury and release of inflammatory mediators. The transition to the chronic phase is characterized by the development of a specific humoral response and a cellular immune response. In both phases, inflammatory mediators act locally or systemically, activating other cells related to the inflammatory process, increasing the initial response to the damaging agent [2,3].

Although these responses are essential for host defense, they must be tightly regulated to prevent de development or aggravation of inflammatory disorders. Thereby, several cellular mediators are secreted to perform essential functions for achieving homeostasis. These mediators may be involved in different pathways, depending on the stimulus and the affected tissue. An inadequate or poorly regulated inflammatory response leads to cellular dysfunction, tissue damage, and impaired repair, commonly observed in numerous inflammatory conditions. Consequently, when the inflammatory response becomes excessive, the use of anti-inflammatory medications is essential to prevent further harm to the body [4,5].

Glucocorticoids and non-steroidal anti-inflammatory drugs (NSAIDs) are standard therapeutic options for controlling inflammation, each modulating distinct cellular signaling cascades. Despite their efficacy, chronic exposure to these drugs often results in adverse effects such as gastrointestinal bleeding, peptic ulceration, hepatotoxicity, nephrotoxicity, and iron-deficiency anemia, ultimately contributing to greater morbidity and mortality [6]. Glucocorticoids, while effective, also come with a range of serious side effects such as immune suppression, osteoporosis, and increased risk of infections, making their long-term use concerning. This widespread, often unsupervised use is concerning, as people of all ages worldwide rely on these medications. Moreover, the high cost of medicines and their toxicity have limited their use and warrant the screening of potential alternative therapies. Given these risks, research has increasingly turned to natural compounds to modulate inflammation, seeking safer alternatives with fewer side effects [7,8].

In this context, medicinal plants represent a rich source of bioactive compounds, whose therapeutic potential for the human body warrants thorough investigation. Among these, polyphenols have emerged as particularly interesting molecules due to their potent anti-inflammatory properties [9,10,11].

Plant-derived polyphenols have consistently demonstrated anti-inflammatory properties in both in vitro and in vivo models, underscoring their potential as therapeutic agents for acute and chronic conditions [12]. In line with this, numerous epidemiological and experimental studies have explored their roles in inflammation and immune modulation. These compounds influence the expression of key pro-inflammatory genes, including cytokines, lipoxygenase, nitric oxide synthases, and cyclooxygenase, while also exerting antioxidant effects through reactive oxygen species (ROS) scavenging, thereby contributing to the modulation of inflammatory signaling [13]. Within this context, *Momordica charantia* (bitter melon or bitter gourd), a member of the Cucurbitaceae family, is distributed across tropical and subtropical regions such as Asia, South America, Africa, the Amazon basin, and the Caribbean [14].

An efficient anti-inflammatory action has been attributed to *M. charantia* in experimental studies, where the plant suppressed LPS-triggered macrophage responses through downregulation of NF-κB activity. In addition, the ethanolic fruit extract was found to reduce LPS-induced nitric oxide, inducible nitric oxide synthase (iNOS), prostaglandin E2 (PGE2), and pro-IL1β expression. It has also been shown that protein expression of COX2 and iNOS in activated mouse macrophages can be inhibited with an ethyl acetate extract of the fruit. In another study, the administration of a freeze-dried fruit powder significantly decreased the protein levels of NF-κB and JNK in a mouse model [15,16].

Despite the extensive investigation of *Momordica charantia* fruits and seeds, its leaves remain largely unexplored, particularly regarding their ability to modulate inflammatory responses [17,18]. In addition to the limited number of studies involving the leaves of *M. charantia*, this part of the plant presents distinctive biochemical features that may differ substantially from the fruit. Leaf extracts are often rich in phenolic acids, flavonoids, and other polar metabolites that play essential roles in the plant’s defense and oxidative stress regulation [19]. Moreover, the use of leaves represents an environmentally sustainable and non-destructive approach, allowing repeated harvesting without compromising the reproductive structures or the ecological balance of the species. Thus, exploring the bioactive potential of *M. charantia* leaves offers both scientific novelty and ecological sustainability [20].

Most available studies are limited to in vitro assays, and there is a clear lack of integrated toxicological and in vivo evaluations, which are essential for translational applications. Moreover, the molecular mechanisms underlying its effects, especially the modulation of inflammation-related gene expression such as TNF-α, MAPK, and NF-κB are poorly characterized.

Therefore, this study is justified by combining phytochemical characterization, OECD-guided toxicological assessment, and both in vitro and in vivo anti-inflammatory models, with gene expression analysis. By simultaneously addressing efficacy, safety, and molecular mechanisms, the work advances the prospecting of *M. charantia* leaf extracts as potential safe and effective anti-inflammatory agents.

## 2. Results

**Figure 1:**
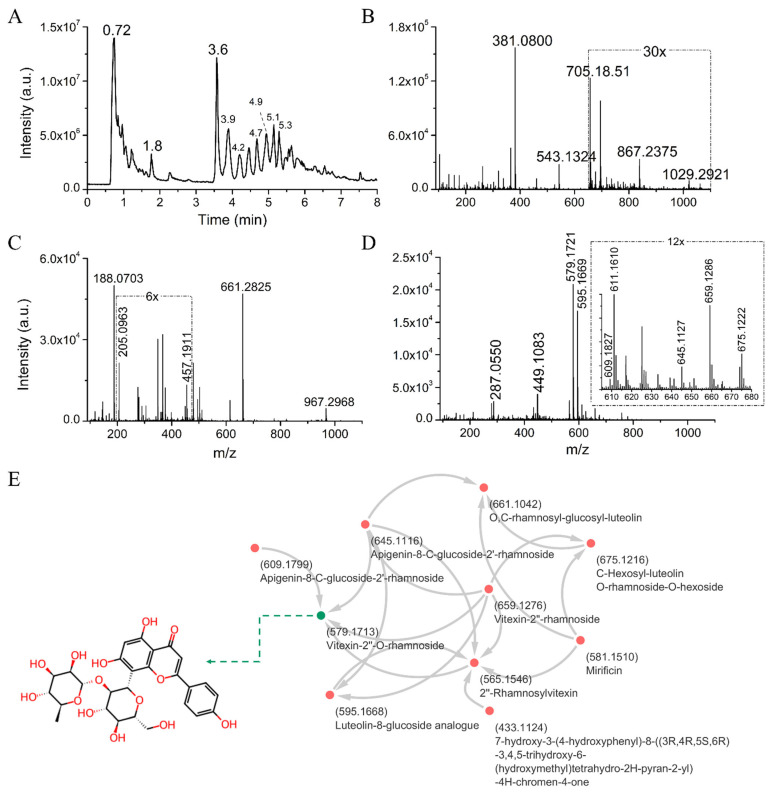
LC-MS/MS fingerprint of *Momordica charantia* hydroethanolic leaf extract (MCHLE). (**A**) Total ion chromatogram showing most intense chromatographic fractions; (**B**–**D**) MS spectra corresponding to 0.7, 3.6 and 4.5–5.3 min, respectively; (**E**) Feature Based Molecular Networking (FBMN) showing ions corresponding to flavonoids clustered together. Dotted boxes represent the magnification of spectrum denoted by numerical indication; FBMN: nodes corresponding to features with mass difference close to zero are represented in green; the numbers in parentheses represent the *m*/*z* of the corresponding feature followed by the name of the matched compound; the putative structures of features with mass difference close to zero are represented.

Momordica charantia hydroethanolic leaf extract (MCHLE) was analyzed by LC-MS/MS and their spectra were submitted to the GNPS2 database in order to identify the detected compounds. Despite the high number of MS/MS spectra acquired for each extract, mainly spectra matching with cosine ≥ 0.85 and a mass difference ≤ 0.005 concerning molecules deposited in the GNPS database were considered for this analysis. In general, the extract chemical profiles showed the putative presence of alkaloids, polysaccharides, anthocyanin, octopamine, ferulate, flavonoid, amino acids and some peptides (Table 1). The total ion chromatogram obtained shows two most intense fractions (at 0.7 and 3.6 min) and a series of less intense fraction between 3.9 and 5.3 min (Figure 1A).

The fraction eluting at 0.7 min exhibited a series of ions consistent with potassium adducts [M+K]+ showing successive neutral losses of hexose units, ranging from two (base peak at *m*/*z* 381.0800) to six (*m*/*z* 1029.2907) (Figure 1B). The presence of polysaccharides in this early chromatographic region is expected due to their high polarity.

The second most intense fraction, at 3.5 min, contained an ion at *m*/*z* 188 previously annotated as L-tryptophan; however, detailed analysis revealed that it represents an in-source fragment derived from the ion at *m*/*z* 661.2823 ([M+H]+), the second most abundant signal in the corresponding spectrum (Figure 1C). The mass balance and fragmentation pattern suggest that this compound is a tryptophan conjugate bound to an oxygenated aromatic aglycone, likely containing one or more sugar-like residues. In-source fragmentation produced both [Trp+H]+ (*m*/*z* 205.0974) and the complementary ion at *m*/*z* 457.1911 ([M–Trp+H]+), whose MS/MS fragments (*m*/*z* 373, 198, 149, 131, 113, and 101) suggest the presence of oxygen-rich substructures typical of saccharide moieties and a nitrogen-containing aromatic core. The accurate mass agreement between precursor and product ions supports this hypothesis, indicating that the molecule is a tryptophan-derived conjugate linked via a labile bond that readily cleaves under ionization. Further experiments will be necessary to confirm its structural nature.

Two additional ions, at *m*/*z* 165.054 and *m*/*z* 166.086, were initially annotated as L-tyrosine and L-phenylalanine, respectively, but were likewise identified as in-source fragments of unknown higher-mass compounds. Finally, fractions eluting between 4.5 and 5.3 min (specifically at 4.5, 4.7, 4.9, 5.2, and 5.3 min) showed base peak ions that, following feature-based molecular networking analysis, clustered together and were all associated at varying levels of confidence with different classes of flavonoids (Figure 1D,E).

Extracted ion chromatograms (XICs) obtained for each structure and identified by UPLC–MS/MS and GNPS analyses showed eight main phytocomponents, with a clear resolution for the MCHLE. Table 1 shows the phytochemical components of the extract and their respective cosines, mass difference, mass, molecular formula, adduct, retention time (RT), and their classification resulting from the ionization process.

**Figure 2:**
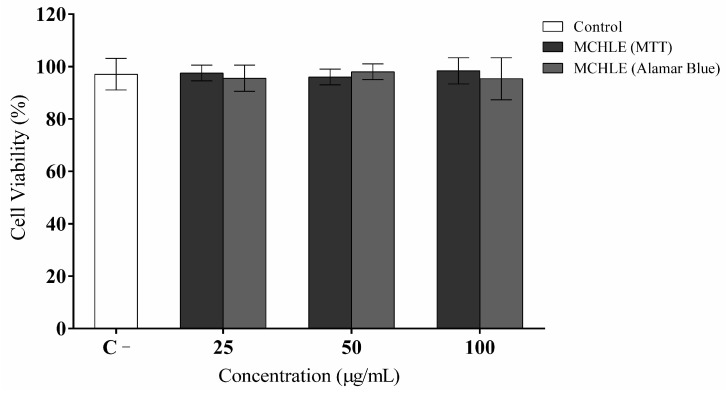
*Momordica charantia* hydroethanolic leaf extract (MCHLE) cell viability effects on RAW 264.7 murine macrophage cells measured by MTT and Alamar Blue^®^ assays. Culture medium DMEM was used as a negative control for cytotoxicity.

Following the phytochemical characterization of MCHLE, cytotoxicity was evaluated using activated RAW 264.7 macrophages. According to the MTT and Alamar Blue^®^ viability assays (Figure 2), treatment with various extract concentrations did not induce cytotoxic effects relative to the control group, indicating good cellular tolerance to MCHLE.

Regarding the acute and repeated oral toxicity assays, both exhibited no behavioral or clinical alterations, signs of intoxication were observed in *Wistar* rats treated with *Momordica charantia* hydroethanolic leaf extract (MCHLE). The biochemical evaluation (Table 2) indicated that most of the parameters remained within normal ranges, supporting the absence of systemic toxicity. However, significant metabolic changes were detected. In the acute toxicity protocol, animals receiving 2000 mg/kg revealed an approximate 50% reduction in glucose levels (152 vs. 73 mg/dL in males), a similar decreased was observed in total cholesterol (58.2 vs. 29 mg/dL). In the repeated dose assay, progressive reductions in glycemia were also observed, with the highest dose group (400 mg/kg) reaching glucose values around 60 mg/dL, nearly 50% lower than the control group (121.7 mg/dL). Similarly, total cholesterol was markedly reduced, up to 53% at 400 mg/kg (24.9 vs. 50.2 mg/dL). Triglyceride concentrations remained stable across groups, while hepatic (ALT, AST, γ-GT) and renal (urea, uric acid, creatinine) markers did not show significant changes, except for mild reductions in urea and uric acid during acute treatment, reinforcing the absence of hepatotoxic or nephrotoxic effects.

The analysis of relative organ weights confirmed these observations, since no statistical differences were found between treated and control groups in either acute or repeated toxicity protocols. The liver, kidneys, spleen, heart, lungs and stomach maintained comparable values across all doses and time points, indicating that MCHLE did not induce hypertrophy, atrophy or organ-specific toxic responses (Table 3).

Oxidative stress parameters further supported the safety profile of the extract (Table 4). In the acute toxicity test, treatment with 2000 mg/kg significantly reduced lipid peroxidation, with MDA levels decreasing by nearly 45% compared to controls (78.1 vs. 43.4 μmol/L). In the repeated dose experiment, a dose-dependent reduction in MDA was also observed, reaching approximately 60% at 400 mg/kg (29.2 vs. 74.2 μmol/L). Antioxidant defenses were preserved or even improved, as glutathione peroxidase (GPx) activity increased progressively with treatment, rising from 5.7 in controls to 11.0 IU/mg protein at 400 mg/kg, while superoxide dismutase (SOD) activity also increased at the highest dose (85.5 vs. 68.3 IU/mg protein). Reduced glutathione (GSH) levels remained stable across all groups, confirming that the extract did not compromise hepatic redox balance.

**Figure 3:**
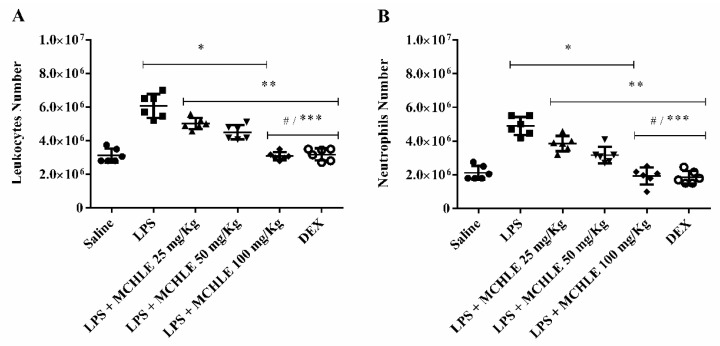
Total leukocyte count (**A**) and neutrophil count (**B**) in the LPS-induced peritonitis model. MCHLE: *Momordica charantia* hydroethanolic leaf extract; Saline: negative control (animals not induced with LPS); LPS: positive control (animals induced with LPS and treated with PBS); DEX: dexamethasone. Mice received an intraperitoneal injection of 100 µL of LPS, followed 1 h later by an intravenous injection of PBS, MCHLE, or DEX. After 4 h, the peritoneal lavage fluid was collected and analyzed for total cell counts. Data are expressed as the mean ± SEM from six animals. Statistical analysis was performed using one-way ANOVA followed by Tukey’s post hoc test. * *p* < 0.05 vs. the saline control group; ** *p* < 0.05 vs. the LPS-only group; # *p* < 0.05 among extract concentrations; *** *p* < 0.05 DEX vs. MCHLE 25 and 50 mg/Kg groups.

In the LPS-induced peritonitis model, MCHLE treatment significantly reduced leukocyte migration into the peritoneal cavity in a dose-dependent manner. As shown in Figure 3A, total leukocyte counts increased from 3.2 ± 0.3 × 10^6^ cells in the saline group to 6.4 ± 0.5 × 10^6^ cells after bacterial LPS stimulation. Treatment with MCHLE lowered these numbers to 4.8 ± 0.4, 4.6 ± 0.3, and 3.1 ± 0.2 × 10^6^ cells at 25, 50, and 100 mg/kg, respectively, values comparable to dexamethasone, used as positive control of anti-inflammatory activity (3.3 ± 0.2 × 10^6^). A similar effect was observed for neutrophils (Figure 3B), where counts rose from 2.2 ± 0.3 × 10^6^ cells in saline controls to 5.1 ± 0.4 × 10^6^ cells in the LPS group, and were significantly reduced by MCHLE to 3.6 ± 0.3, 3.2 ± 0.4, and 2.1 ± 0.3 × 10^6^ cells at 25, 50, and 100 mg/kg, respectively, again approaching values obtained with dexamethasone (2.2 ± 0.4 × 10^6^). These findings confirm that MCHLE effectively suppresses leukocyte, particularly neutrophil, infiltration during acute inflammation.

**Figure 4:**
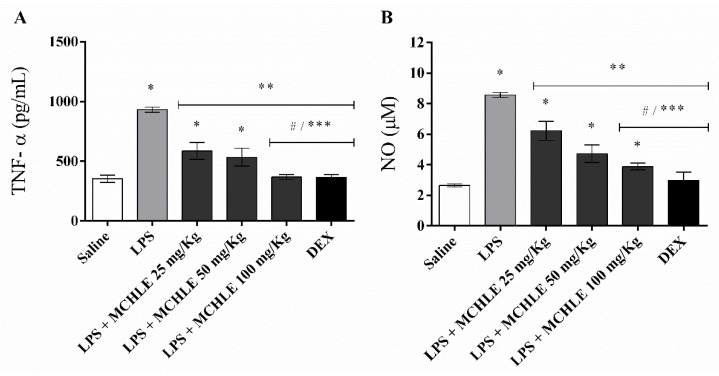
LPS-induced cytokine (**A**) and nitric oxide (**B**) production in C57BL/6 mice. Groups included Saline (negative control, non-stimulated animals), LPS (positive control, animals induced with LPS and treated with PBS), MCHLE (*Momordica charantia* hydroethanolic leaf extract, different concentrations), and DEX (dexamethasone). Data are expressed as the mean ± SD of cytokine levels (pg/mL) or nitric oxide concentrations (µM) in the peritoneal fluid, with NO quantified using the Griess reagent. One-way ANOVA followed by the post hoc Tukey’s test. * *p* < 0.05 vs. the saline control group; ** *p* < 0.05 vs. the LPS-stimulated only; # *p* < 0.05 between the concentrations of the extracts; *** *p* < 0.05 DEX vs. MCHLE 25 and 50 mg/Kg groups.

MCHLE treatment also modulated inflammatory mediators in the peritonitis model. As shown in Figure 4A, TNF-α levels increased from 386.2 ± 30.7 pg/mL in the saline group to 931.0 ± 22.3 pg/mL after LPS stimulation. MCHLE administration reduced these levels in a dose-dependent manner, reaching 586.9 ± 66.5 pg/mL at 25 mg/kg, 534.3 ± 70.3 pg/mL at 50 mg/kg, and 367.7 ± 20.1 pg/mL at 100 mg/kg, the latter comparable to dexamethasone (366.7 ± 20.5 pg/mL). In parallel, nitric oxide concentrations (Figure 4B) rose from 2.64 ± 0.10 µM in the saline group to 8.57 ± 0.14 µM in the LPS control, whereas MCHLE treatment lowered NO levels to 6.22 ± 0.56 µM, 4.93 ± 0.45 µM, and 3.89 ± 0.22 µM at 25, 50, and 100 mg/kg, respectively, with values similar to dexamethasone (3.31 ± 0.52 µM). These results suggest the ability of MCHLE to attenuate cytokine release and NO production during acute inflammation.

**Figure 5:**
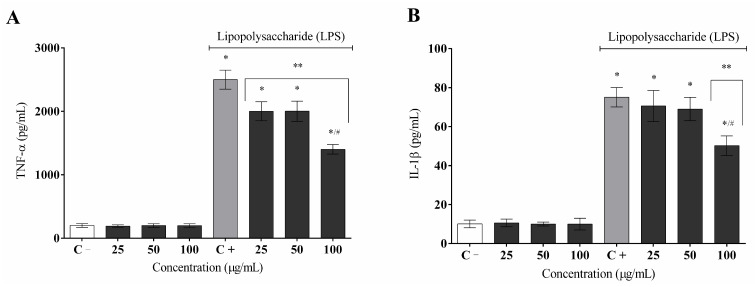
Effect of *Momordica charantia* hydroethanolic leaf extract (MCHLE) on the TNF-α (**A**) and IL-1β (**B**) cytokine release. The cytokine content was released in RAW 264.7 cells and stimulated by LPS after 24 h. Release of cytokines was quantified by ELISA. Data represent the mean ± SEM from three independent experiments. One-way ANOVA followed by the post hoc Tukey’s test. * *p* < 0.05 vs. the negative control group; ** *p* < 0.05 vs. the LPS-stimulated cells; # *p* < 0.05 between the concentrations of the extracts.

In LPS-stimulated RAW 264.7 macrophages, MCHLE significantly inhibited the release of pro-inflammatory mediators in a concentration-dependent manner (Figure 5). Regarding TNF-α, basal levels in the non-stimulated control were 200.1 ± 30.0 pg/mL, whereas LPS increased secretion to 2500.0 ± 150.0 pg/mL. Treatment with MCHLE reduced these values to 2000.5 ± 150.0 pg/mL at 25 µg/mL, 2000.9 ± 160.0 pg/mL at 50 µg/mL, and 1400.2 ± 75.0 pg/mL at 100 µg/mL, indicating a marked inhibition at the highest concentration (Figure 5A). For IL-1β, basal levels were 10.1 ± 2.0 pg/mL, and stimulation with LPS raised secretion to 75.1 ± 5.0 pg/mL. MCHLE reduced cytokine levels to 70.5 ± 8.0 pg/mL at 25 µg/mL, 69.0 ± 6.0 pg/mL at 50 µg/mL, and 50.2 ± 5.0 pg/mL at 100 µg/mL, again showing a clear dose-dependent effect (Figure 5B).

**Figure 6:**
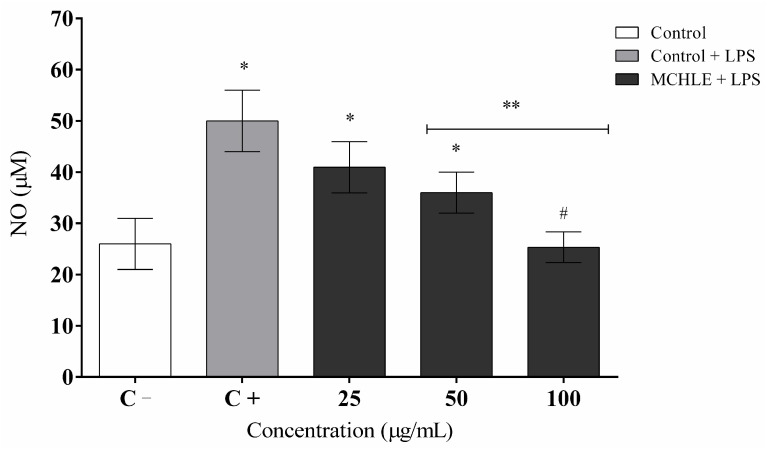
Inhibitory effects of *Momordica charantia* hydroethanolic leaf extract (MCHLE) on LPS-stimulated nitric oxide (NO) production in RAW 264.7 macrophages. The nitric oxide (NO) levels in the culture medium were quantified using the Griess reagent. Data are expressed as the mean ± SEM from three independent experiments. Statistical analysis was performed using one-way ANOVA followed by Tukey’s post hoc test. * *p* < 0.05 vs. the negative control group; ** *p* < 0.05 vs. the LPS-stimulated cells; # *p* < 0.05 among extract concentrations.

Regarding nitric oxide, basal production was 26.0 ± 5.0 µM, while LPS stimulation increased levels to 50.0 ± 6.0 µM. MCHLE significantly reduced NO generation to 40.9 ± 5.0 µM at 25 µg/mL, 35.9 ± 4.0 µM at 50 µg/mL, and 25.3 ± 3.0 µM at 100 µg/mL, restoring values close to the non-stimulated control (Figure 6).

**Figure 7:**
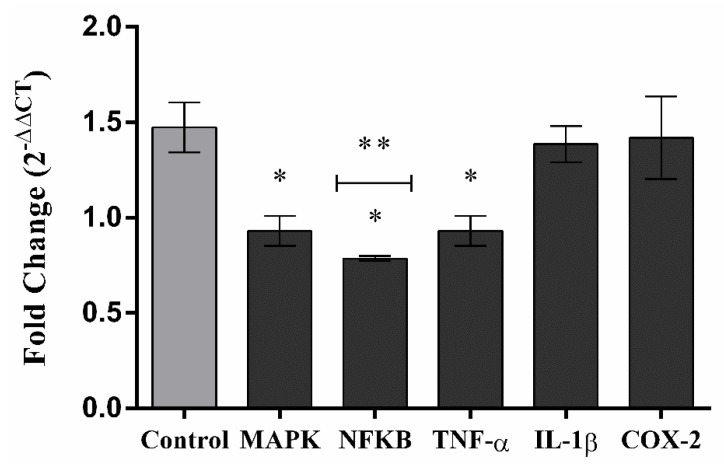
Inflammation-related gene expression in RAW264.7 macrophages cells. Cells were treated with 100 µg/mL of *Momordica charantia* hydroethanolic leaf extract (MCHLE). Cells from control group were plated with DMEM medium only. After 24 h, the expression levels of MAPK; NFKB; TNF-α; IL-1β and COX-2 genes were quantified by real-time qPCR. The endogenous genes GAPDH and β-ACTIN were used as a real-time qPCR internal control. The expression gene was calculated by the 2-ΔΔCT method. Data represent the mean ± SEM from three independent experiments. One-way ANOVA followed by the post hoc Tukey’s test. * *p* < 0.05 vs. the LPS-stimulated cells (Control Group); ** *p* < 0.05 between the Inflammation-related genes.

Consistent with the reduction in cytokine release and nitric oxide production, MCHLE also downregulated the expression of key inflammatory genes in LPS-stimulated RAW 264.7 macrophages (Figure 7). The extract decreased TNF-α and MAPK transcripts in a concentration-dependent manner, while the most pronounced inhibition was observed for NF-κB, whose expression was strongly suppressed at 100 µg/mL, approaching basal levels. These results indicate that the extract not only modulates the release of inflammatory mediators but also targets upstream transcriptional regulators, with NF-κB being the most sensitive to inhibition.

## 3. Discussion

Nonsteroidal anti-inflammatory drugs (NSAIDs) are among the most widely used classes of medications worldwide, particularly for the treatment of inflammation, pain, edema, osteoarthritis, and other conditions [6]. A key factor behind their widespread use is that they are available as over-the-counter drugs; however, their inappropriate and indiscriminate use has raised concerns regarding severe side effects, such as gastric ulcers, bleeding, and renal dysfunction, as well as their overall impact on public health. In this context, medicinal plants emerge as a viable alternative to counteract inflammatory processes, being culturally accepted, especially in communities with limited access to conventional NSAID treatments [21].

Our findings support the strategic exploration of plant-based anti-inflammatory approaches as complementary or alternative options to NSAIDs. In this context, we identify the hydroethanolic leaf extract of *Momordica charantia* (MCHLE) as a safe, multi-target modulator of acute inflammation, warranting closer examination of its mechanisms and translational potential.

The present study demonstrates that MCHLE combines a favorable toxicological profile with robust anti-inflammatory activity across in vivo and in vitro complementary models. In vivo (LPS-induced peritonitis), MCHLE reduced total leukocyte and neutrophil influx and lowered TNF-α and NO levels to values comparable to dexamethasone; in vitro (LPS-stimulated RAW 264.7), it produced dose-dependent inhibition of TNF-α, IL-1β, and NO. At the transcriptional level, MCHLE downregulated TNF-α and MAPK and strongly suppressed NF-κB, indicating engagement of upstream regulators consistent with the mediator decreases observed. Taken together, these converging effects across biochemical, cellular, and transcription levels support a coherent, multi-level mechanism of action.

LC–MS/MS profiling identified phenylpropanoid derivatives (ferulate/isoferulate), flavonoids (vitexin-2-O-rhamnoside, flavones, isoflavones glycosides), and bioactive amino acids (tyrosine, tryptophan). Phenylpropanoids and flavonoids are well known to suppress NF-κB activation and modulate MAPK cascades; for example, vitexin attenuates macrophage inflammation via NF-κB inhibition, whereas ferulate derivatives influence oxidative stress and MAPK signaling [21,22,23]. Therefore, it is plausible that these molecules contribute synergistically to the pharmacological activity observed in vivo and in vitro. This phytochemical fingerprint provides a plausible mechanistic basis for MCHLE’s dual antioxidant/anti-inflammatory profile and suggests that complementary actions among constituents may underpin the breadth of activity observed. Future work should focus on targeted quantification and isolation of these compounds to confirm their mechanistic roles.

Toxicological assessment indicated safety under acute (2000 mg/kg) and repeated dosing (≤400 mg/kg), without hepatic, renal, or behavioral alterations. Concomitantly, MCHLE lowered blood glucose and total cholesterol across protocols, suggesting ancillary metabolic benefits that may intersect with inflammatory control, given the established links between dysmetabolism, redox imbalance, and immune activation [24]. Although a significant reduction in indirect bilirubin (IB) was observed in the high-dose group (400 mg/kg), all other hepatic biomarkers remained within normal physiological ranges, excluding any indication of hepatotoxicity. This decrease may reflect an increase in bilirubin conjugation efficiency or antioxidant-mediated modulation of hepatic enzymes, as reported for polyphenolic compounds such as ferulic acid and vitexin derivatives. Therefore, the reduction in IB likely represents an adaptive metabolic response rather than an adverse effect [25,26]. These data position MCHLE as a candidate not only for anti-inflammatory effects but also for broader homeostatic support under inflammatory stress.

Our findings are consistent with prior reports in LPS-stimulated RAW 264.7 macrophages showing that *M. charantia* reduces classical pro-inflammatory transcripts (IL6, TNF-α, IL1β, COX2, iNOS) [24,25,26,27,28]. We extend those observations by demonstrating concurrent suppression of NF-κB at the transcriptional level together with reductions in cytokines and NO, aligning upstream pathway modulation with functional readouts. Minor differences in magnitude across studies are expected and likely reflect extraction procedures, phytochemical composition, and cell-context variability [24,29].

Metabolic reprogramming may contribute to MCHLE’s anti-inflammatory action. Prior data indicate that *M. charantia* attenuates LPS-induced glycolytic shifts by downregulating GLUT1 and HK2, thereby blunting Warburg-like metabolism [24]. This mechanism is compatible with our observation of reduced systemic glucose and with the dampening of NO and cytokine outputs, suggesting that MCHLE may temper inflammatory signaling in part by limiting glycolytic drive and the associated redox pressure [30,31]. Targeted metabolomics and flux analyses will be valuable to confirm this axis.

Independent studies have also described hepatoprotective actions of *M. charantia* preparations, including mitigation oxidative stress and modulation of apoptotic and inflammatory pathways in toxin-induced liver injury models [32,33,34]. Although our in vivo model addressed acute peritonitis, these findings align with prior reports of *M. charantia*’s hepatoprotective and antioxidants effects, reinforcing the multi-component, synergistic nature of MCHLE.

MCHLE markedly decreased hepatic lipid peroxidation (TBARS/MDA) while enhancing endogenous antioxidant defenses (GPx, SOD). By weakening the oxidative-inflammatory amplification loop, this redox control likely contributes to the observed reductions in TNF-α and NO and to the restraint of leukocyte recruitment in vivo [35,36]. The alignment between antioxidant enzyme upregulation and mediator suppression points to a coordinated attenuation of both oxidative and inflammatory nodes [37].

In line with the redox findings, MCHLE reduced NO at 25–100 µg/mL in LPS-stimulated macrophages, consistent with literature indicating that *M. charantia* enhances antioxidant defenses (SOD) and sustains redox homeostasis [31]. Given the central role of iNOS-derived NO in LPS responses, the combination of NO attenuation and NF-κB suppression suggests complementary actions on both enzymatic drivers and upstream transcriptional control [38].

Overall, MCHLE acts as a phytocomplex with synergistic activities, simultaneously enhancing endogenous antioxidant defenses and attenuating canonical inflammatory pathways (NF-κB/MAPK). This dual action resulted in consistent reductions in cytokines and NO, as well as limited leukocyte and neutrophil recruitment in vivo. Notably, the profile observed suggests a balanced immunomodulatory effect rather than broad immunosuppression, a characteristic that strengthens its potential safety and translational relevance [39,40].

Future investigations should apply activity-guided fractionation to identify the key bioactive constituents, establish precise dose-response relationships, and characterize the pharmacokinetic and pharmacodynamic profiles, including bioavailability and target engagement. Moreover, studies employing chronic and comorbidity-relevant models are needed to complement the acute peritonitis findings.

Although the present study successfully identified important metabolites in the hydroethanolic leaf extract of *M. charantia*, absolute quantification was not performed. Future studies should include quantitative LC–MS/MS analyses using analytical standards to establish direct correlations between metabolite concentrations and bioactivity. Additionally, evaluating the biological effects of isolated compounds such as octopamine, ferulate, and vitexin-2-O-rhamnoside will be essential to confirm their individual pharmacological contributions.

Finally, chemical standardization using marker compounds like vitexin-2-O-rhamnoside and ferulate derivatives will be crucial to ensure batch-to-batch reproducibility and to support the development of safe and effective formulations. Overall, these findings position MCHLE as a promising phytochemical source for development of anti-inflammatory agents with potential metabolic benefits.

## 4. Materials and Methods

### 4.1. Plant Material and Extract Preparation

*Momordica charantia* leaves were collected in Parnamirim, Rio Grande do Norte, Brazil (5°56′16″ S, 35°16′27″ W) in September 2022, under approval from the Authorization and Information System in Biodiversity (SisBio) and the National System for the Management of Genetic Heritage and Associated Traditional Knowledge (SisGen). The plant material was taxonomically identified by Dr. Jomar Gomes Jardim at the Herbarium of the Department of Botany and Zoology, Federal University of Rio Grande do Norte (Natal, RN, Brazil), and deposited under registration number 00010853.

After collection, the leaves were cleaned and air-dried at 40 °C for 48 h. The dried material was grounded to a particle size of 0.5–1.0 mm and stored in amber containers until extract preparation. A total of 300 g of powdered leaves was macerated in 1.5 L of a 50:50 (*v*/*v*) ethanol-water mixture for four days at room temperature. The filtrate was concentrated using rotary evaporation and subsequently freeze-dried, yielding the hydroethanolic leaf extract of *M. charantia* (MCHLE).

### 4.2. Phytochemical Analysis by Ultrafast Liquid Chromatography Coupled to Mass Spectrometry (LC–MS/MS)

For LC-MS/MS analysis, the extract was reconstituted in methanol (5 mg/mL), centrifuged at 13,000 rpm for 30 min, and filtered through a 0.22 µm membrane. The resulting supernatant was stored at −20 °C and subsequently, the extract was diluted in 50% acetonitrile before injection into the LC–MS/MS system.

Chromatographic separation was carried out on an Eksigent UltraLC 110-XL system (AB Sciex, Framingham, MA, USA) equipped with a Kinetex C18 column (2.6 µm, 100 Å, 50 × 2.1 mm) coupled to a TripleTOF 5600+ mass spectrometer (AB Sciex, Framingham, MA, USA). The column was equilibrated with 5% acetonitrile/0.1% formic acid for 5 min, after which 2 µL of sample was injected. Elution was performed with a linear gradient of acetonitrile (5–95%, containing 0.1% formic acid) over 10 min at a flow rate of 0.4 mL/min, with the column temperature maintained at 40 °C. Mass spectrometric detection was conducted in positive IDA mode (*m*/*z* 100–1800), with source temperature of 650 °C. Fragmentation was acquired for ions between *m*/*z* 100 and 1250, charge states 1–3, and intensities above 1000 counts. Additional acquisition parameters included: cycle time, 900 ms; pulser frequency, 15.392 kHz; accumulation time, 250 ms; curtain gas, 15; ion source gas 1, 50; ion source gas 2, 45; and ion spray voltage, 5500 V. Blank runs were acquired for quality control. Instrument calibration was performed before analysis and after every five injections using AB Sciex calibration solution (sodium iodide, 2 µg/µL; cesium iodide, 50 ng/µL in 50/50 2-propanol/water), ensuring mass accuracy within ~0.5 ppm.

Raw acquisition files (.WIFF) were converted to .mzXML format with MSConvert (ProteoWizard 3.0) and processed in GNPS (Global Natural Products Social Molecular Networking; [http://gnps.ucsd.edu] (accessed on 27 July 2025) using the Molecular-Library Search-V2 workflow (release\_14). Data pre-processing involved removal of precursor-related peaks (−17 Da), MS/MS window filtering to retain the top 6 peaks within 50 Da, and clustering via MS-Cluster (precursor mass tolerance 0.02 Da, fragment ion tolerance 0.1 Da) to generate consensus spectra. Spectra supported by fewer than two MS/MS events were discarded. GNPS library matching was conducted under the same filtering parameters, requiring a cosine score ≥ 0.85 and at least four matched peaks for spectral assignment. The cosine score, based on a normalized dot product, reflects spectral similarity, with 1 indicating identical profiles and 0 denoting no similarity.

### 4.3. Cell Culture and Animals

Murine macrophages (RAW 264.7, ATCC^®^ TIB-71™, Rockville, MD, USA) were employed in vitro. Cultures were grown in DMEM supplemented with 10% FBS and antibiotics (penicillin, 5000 IU/mL; streptomycin, 5000 µg/mL) at 37 °C, in a humidified incubator with 5% CO_2_.

For the in vivo assays, Wistar rats (250–300 g, two months old, both sexes) and male C57BL/6 mice (25–30 g) were obtained from the Vivarium of the Health Sciences Center, Federal University of Rio Grande do Norte (Natal, RN, Brazil). The animals were housed in collective cages (n = 5) under controlled environmental conditions, with a 12 h light/dark cycle and a constant temperature of 22 ± 2 °C, and had free access to food and water (*ad libitum*). All experimental procedures were approved by the Ethics Committee on Animal Use of the Federal University of Rio Grande do Norte (Protocol No. 254.021/2021).

### 4.4. Cell Viability and Cytotoxicity Assays

The cytotoxic potential of MCHLE was evaluated in RAW 264.7 macrophages using the MTT reduction assay. Cells (1 × 10^5^ per well) were seeded in 96-well plates and allowed to adhere for 24 h at 37 °C. Subsequently, they were treated in triplicate with MCHLE at concentrations of 25, 50, and 100 µg/mL. After the incubation period, 100 µL of MTT solution (5 mg/mL) was added to each well, followed by a 4 h incubation at 37 °C. The supernatant was then carefully removed, 100 µL of dimethyl sulfoxide (DMSO) was added to solubilize the formazan crystals, and absorbance was measured at 570 nm using a microplate reader (Epoch, BioTek, Winooski, VT, USA). Untreated cells cultured in DMEM served as the negative control.

Cell viability was also determined by the Alamar Blue^®^ assay. For this, RAW 264.7 macrophages (1 × 10^5^/well) were seeded into 96-well plates, pre-incubated for 24 h, and then treated with MCHLE (25, 50, or 100 µg/mL) for an additional 24 h at 37 °C. Following treatment, Alamar Blue^®^ reagent corresponding to 10% of the well volume was added and plates were incubated for 4 h under standard conditions (37 °C, 5% CO_2_). Fluorescence reduction was quantified by measuring absorbance at 570 and 600 nm in a microplate reader (Epoch, BioTek, Winooski, VT, USA). DMEM-cultured cells were used as negative controls, following the method described by Luz et al. [41].

### 4.5. Acute Toxicity

The acute toxicity assay was conducted in accordance with the guidelines of the Brazilian National Health Surveillance Agency [42] and the Organization for Economic Cooperation and Development [43]. Male Wistar rats (60 days old) were randomly assigned to two groups (n = 3): one group received a single oral dose of MCHLE (2000 mg/kg), while the control group received distilled water only (not exceeding 1 mL/100 g body weight). Both treatments were administered by oral gavage. Following administration, animals were monitored for signs of systemic toxicity, including vocal tremors, piloerection, hyperactivity, tremors, abdominal cramps, diarrhea, and mortality. Observations were recorded every 3 h during the first 12 h, every 6 h on the following day, and at least twice daily thereafter. Water and food intake were measured at two-day intervals, and body weight was recorded weekly. On the 14th day, animals were anesthetized with a xylazine–ketamine mixture (1:1), euthanized, and subjected to laparotomy for blood collection via cardiac puncture to evaluate biochemical parameters. Subsequently, a macroscopic necropsy was performed to determine relative organ weights (liver, kidney, spleen, lung, heart, and stomach). Liver samples were rinsed with 0.9% NaCl for subsequent analysis of antioxidant activity, as described by Batista et al. [44].

### 4.6. Repeated Dose Oral Toxicity 28 Days

This procedure was conducted using 60-day-old Wistar rats of both sexes, distributed into four groups in accordance with Organization for Economic Cooperation and Development guidelines [45]. Animals received oral gavage treatments with MCHLE at doses of 100, 200, or 400 mg/kg for 28 consecutive days, as described by Batista et al. [44]. The control group received distilled water only. Throughout the experimental period, animals were observed for signs of toxicity and mortality. Behavioral and physiological parameters were recorded every 3 h during the first 12 h, every 6 h on the following day, and at least twice daily thereafter. Water and food intake were monitored at two-day intervals, and body weight was measured weekly. At the end of the 28-day treatment, animals were anesthetized with a xylazine–ketamine mixture (1:1) and euthanized. Blood samples were collected via cardiac puncture for biochemical analysis, and organs (liver, kidney, spleen, lung, heart, and stomach) were excised for macroscopic examination and relative weight determination. Liver tissues were rinsed with 0.9% NaCl for subsequent assessment of antioxidant activity, as described by Batista et al. [44].

### 4.7. Evaluation of Biochemical Parameters and Preparation of Hepatic Homogenate

Serum samples collected individually from each rat (n = 3 per group) were used to determine biochemical parameters, including glucose, triglycerides, cholesterol, alanine aminotransferase (ALT), aspartate aminotransferase (AST), bilirubin, urea, creatinine, total protein, albumin, and amylase levels. These analyses were performed using commercial Labtest^®^ kits, following the manufacturer’s instructions, in a LabMax Plenno automatic analyzer (Lagoa Santa, MG, Brazil).

Liver tissue samples (1 g) were homogenized in ice-cold 20 mM potassium phosphate buffer (pH 7.4) using a Potter–Elvehjem homogenizer for 20 s to obtain a 10% (*w*/*v*) homogenate. The homogenates were centrifuged at 4 °C for 4 min, and the resulting supernatant was used to determine thiobarbituric acid reactive substances (TBARS), reduced glutathione (GSH) levels, and the activities of antioxidant enzymes, including superoxide dismutase (SOD) and glutathione peroxidase (GPx).

### 4.8. Oxidative Stress Analyses

The concentration of thiobarbituric acid reactive substances (TBARS, mmol/L) was determined using the colorimetric method described by Yagi [46]. The reduced glutathione (GSH) content in the liver homogenate (mmol/L) was quantified according to the procedure proposed by Beutler, Duron, and Kelly [47]. The activities of superoxide dismutase (SOD) and glutathione peroxidase (GPx) were measured at 510 nm and 340 nm, respectively, using commercial kits (Randox Laboratories Ltd., Crumlin, UK).

### 4.9. Leukocyte Migration into Peritoneal Cavity and Cytokine Dosage

Male C57BL/6 mice were randomly divided into six groups (n = 6) as follows: Group 1, negative control (PBS only); Group 2, positive control (LPS only); Groups 3, 4, and 5, treated with MCHLE at doses of 25, 50, and 100 mg/kg, respectively; and Group 6, treated with dexamethasone (2 mg/kg). Acute inflammation was induced in Groups 2–6 by intraperitoneal injection of lipopolysaccharide (LPS, E. coli O55:B5 strain, 2 µg/mL). After 15 min, MCHLE (25, 50, or 100 mg/kg) or dexamethasone (2 mg/kg) was administered intravenously to the respective treatment groups. Four hours after induction, the mice were anesthetized with a xylazine-ketamine mixture (1:1) and euthanized. The peritoneal cavity was washed with 2 mL of 0.5% saline solution containing 1 mM EDTA, and the peritoneal lavage fluid was collected. Total leukocyte counts were determined using a hemocytometer, and differential polymorphonuclear leukocyte (PMN) counts were obtained from cytospin preparations stained with hematoxylin and eosin. Peritoneal fluid samples from each group were stored at −80 °C for subsequent analysis of TNF-α and IL-1β levels by enzyme-linked immunosorbent assay (ELISA) and for nitric oxide (NO) quantification.

### 4.10. Cytokine Measurement (TNF-α and IL1-β)

RAW 264.7 macrophages (1 × 10^5^ cells/well) were seeded in 96-well plates and pre-stimulated for 1 h with LPS (2 µg/mL in DMEM). Cells were then treated with MCHLE at concentrations of 25, 50, or 100 µg/mL. After 24 h of incubation, culture supernatants were collected and analyzed for TNF-α and IL-1β using commercial ELISA kits (eBioscience, San Diego, CA, USA) according to the manufacturer’s guidelines. Absorbance was measured at 450 nm in a microplate reader (Epoch, BioTek, Winooski, VT, USA). Assays were carried out in triplicate. LPS-stimulated cells without extract served as positive controls, while untreated cells were used as negative controls.

For the in vivo assay, peritoneal fluid samples were collected from LPS-induced mice. TNF-α concentrations were quantified using the same ELISA kit (eBioscience), following the manufacturer’s protocol. Measurements were performed in triplicate, and absorbance was recorded at 450 nm.

### 4.11. Measurement of Nitric Oxide (NO) Production

RAW 264.7 macrophages (1 × 10^5^ cells/well) were seeded in 96-well plates and stimulated with LPS (2 µg/mL in DMEM) for 1 h before treatment with MCHLE at 25, 50, or 100 µg/mL. After 24 h, culture supernatants were collected and nitric oxide (NO) production was quantified using the Griess reaction. Briefly, 40 µL of supernatant was mixed with Griess reagent, and absorbance was recorded at 545 nm with a microplate reader (Epoch, BioTek, Winooski, VT, USA). Controls consisted of LPS-stimulated cells without extract (positive control) and unstimulated cells (negative control).

In the in vivo model of LPS-induced inflammation, peritoneal exudates were collected from each group and analyzed for NO content using the same Griess method. Aliquots of 100 µL of peritoneal fluid were treated with Griess reagent, and absorbance was measured at 545 nm. All determinations were performed in triplicate using the same microplate reader (Epoch, BioTek, Winooski, VT, USA).

### 4.12. Gene Expression Analysis

#### 4.12.1. RNA Extraction and cDNA Production

RAW 264.7 cells (2.4 × 10^6^ cells/well) were plated in 06 well plates in DMEM medium supplemented with 10% FBS and induced with LPS (O55:B5 strain). After 1h, 1.5 mL of DMEM medium containing MCHLE at 100 μg/mL were added in each well. 48h later, medium was removed, and the cells were lysed with Trizol and RNA extraction was performed according to PureLink^®^ RNA Mini Kit extraction instructions. After obtaining the RNA samples, total RNA was treated with TURBO DNase I kit (Ambion, Austin, TX, USA). After digestion, RNA was tested by the DNA presence. Only the total RNA that did not have any DNA was used for obtain cDNA by reverse transcription. For this purpose, it was performed in a final volume of 20 μL, using 10–13 μL of total RNA (3 μg) and 7–10 μL of 2 times RT Master Mix of the High Capacity cDNA Reverse Transcription Kit (Applied Biosystems, Foster City, CA, USA), according to the manufacturer’s instructions. The cDNA resulting from this reaction was quantified for absorbance ratio analysis between 260 nm and 280 nm (A260/A280) using the NanoDrop 2000 Spectrophotometer (Thermo Fisher Scientific—NanoDrop products, Wilmington, DE, USA).

#### 4.12.2. Inflammation-Related Gene Expression Quantification

Expression of genes related to inflammation development were quantified by Real-Time (RT) qPCR on 7500 Standard equipment (Applied Biosystems, Foster City, CA, USA) and the ABI inventoried TaqMan^®^ Gene Expression Assays, whose genes are listed in Supplementary Material. 20 μL final volume was used. For each sample analyzed, 1 μL of ABI inventoried TaqMan^®^ Gene Expression Assays, 10 μL of TaqMan^®^ 2-fold PCR Master Mix and 9 μL of cDNA at 20 ng/μL were used. The conditions for cycling amplification were: hold step 50 °C for 2 min, denaturation at 95 °C for 10 min, followed by 40 cycles of a two-step program (denaturation at 95 °C for 15 s, and annealing/extension at 60 °C per 1 min). RT-qPCR data were analyzed by the ΔΔCT method. A line, the so-called threshold, was passed through the geometric portions of the amplification curves. The number of cycles where the threshold line crosses the curves is called CT. The ΔCT was obtained as the difference between the CTs for 18S RNA and the CT for a gene of interest (corresponding to the average of triplicate or duplicate wells from the same cDNA), at each time studied. The lowest mRNA average obtained from the control cells was set as a calibrator and subtracted from each ΔCT value, thus obtaining the ΔΔCT. This value was used as the negative exponential of base 2 (2-ΔΔCT).

To analyses the temporal gene expression of each group, as well as the comparison between control and treated groups, the log values of gene expression were averaged (n = 3–6 flasks from at least two independent experiments) and plotted as the mean ± SEM relative to the lowest value of control group in RAW 264.7 cells.

### 4.13. Statistical Analysis

Data were expressed as the mean ± SEM and analyzed using one-way ANOVA followed by Tukey’s post hoc test. Normality of data distribution was verified using the Shapiro–Wilk test, and homogeneity of variances was assessed using Levene’s test. Statistical analyses were performed in GraphPad Prism version 6.0 for Windows (GraphPad Software, San Diego, CA, USA). A value of *p* < 0.05 was considered statistically significant.

## 5. Conclusions

The present study demonstrates that the *Momordica charantia* hydroethanolic leaf extract (MCHLE) is safe under both acute and repeated oral administration, while exerting significant pharmacological effects. In vivo, the extract reduced glucose and cholesterol levels, inhibited leukocyte and neutrophil infiltration, and suppressed TNF-α and nitric oxide production in the peritonitis model. Complementary in vitro assays with RAW 264.7 macrophages confirmed these findings, revealing dose-dependent inhibition of TNF-α, IL-1β, and NO secretion, along with strong downregulation of MAPK and, most notably, NF-κB gene expression. Together, these results indicate that MCHLE attenuates the release of inflammatory mediators and targets upstream molecular pathways, suggesting its potential as a safe and effective anti-inflammatory candidate worthy of further pharmacological and toxicological evaluation.

## Figures and Tables

**Table 1:** Phytocomponents identified in *Momordica charantia* hydroethanolic leaf extract (MCHLE) by LC–MS/MS analyses.

Phytocomponents Matched with GNPS Database	Cosine	Mass-Diff	Mass	Molecular Formula	Adduct	RT (min)	Classification	Shared Peaks
Trigonelline	0.83	0.000	138.055	C_7_H_7_NO_2_	[M+H]+	0.7	Alkaloid	5
Polysaccharide	MA	0.001	867.2413	C_30_H_52_O_26_	[M+K]+	0.8	Polysaccharide	MA
DL-Octopamine	0.95	0.000	136.076	C_8_H_11_NO_2_	[M+H]+	1.0	Biogenic amine	5
L-Tyrosine *	0.94	0.001	165.054	C_9_H_8_O_3_	[M+H]+	1.0	Amino acid	5
L-(+)-norleucine	0.98	0.001	132.102	C_6_H_13_NO_2_	[M+H]+	1.0	Amino acid	6
Ciclo(His-Pro)	MA	0.001	235.1182	C_11_H_14_N_4_O_2_	[M+H]+	1.1	Ciclic dipeptide	MA
N-Fructosyl tyrosine	0.78	0.001	344.133	C_15_H_21_NO_8_	[M+H]+	1.1	Glycated amino acid	10
Phenylalanine *	0.96	0.000	166.086	C_9_H_11_NO_2_	[M+H]+	1.2	Amino acid	5
Fru-Gly-Leu/Ile	0.73	0.001	351.177	C_14_H_26_N_2_O_8_	[M+H]+	2.1	Dipeptide	11
Deoxycarnitine	0.96	0.057	146.06	C_7_H_15_NO_2_	[M+H]+	3.6	Carnitine derivative	5
L-Tryptophan *	0.97	0.000	188.07	C_11_H_9_N_1_O_2_	[M−NH3+H]^+^	3.6	Amino acid	7
Adenosine, 5_-S-methyl-5_-thio-	0.93	0.000	298.097	C_11_H_15_N_5_O_3_S	[M+H]+	3.9	Purine nucleos(t)ides	11
Ferulate/isoferulate	0.89	0.000	177.054	C_10_H_8_O_3_	[M+H]+	4.2	Phenolic acid	7
O,C-rhamnosyl-glucosyl-luteolin	0.75	66.104	661.104	NA	[M+H]+	4.5	Flavones	9
Vitexin-2″-rhamnoside	0.88	79.957	659.128	C_27_H_30_O_14_	[M+H]+	4.6	Flavones	11
Riboflavin	0.83	0.000	377.146	C_17_H_20_N_4_O_6_	[M+H]+	4.7	Pteridine alkaloids	12
Apigenin-8-C-glucoside-2′-rhamnoside	0.83	65.941	645.112	C_27_H_30_O_14_	[M+H]+	4.7	Flavones	12
C-Hexosyl-luteolin O-rhamnoside-O-hexoside	0.74	81.878	675.122	NA	[M+H]+	4.7	Flavones	8
Mirificin	0.78	31.991	581.151	C_26_H_28_O_13_	[M+H]+	4.9	Isoflavone glycoside	9
7-O-beta-glucopyranosyl-4′-hydroxy-5-methoxyisoflavone	0.91	0.001	447.13	C_22_H_22_O_10_	[M+H]+	5.0	Isoflavone glycoside	5
Diprotin A	0.76	0.000	342.2386	C_17_H_31_N_3_O_4_	[M+H]+	5.1	Dipeptide	MA
Vitexin-2″-O-rhamnoside	0.96	0.001	579.171	C_27_H_30_O_14_	[M+H]+	5.1	Flavones	15
2″-Rhamnosylvitexin	0.93	14.015	565.155	C_27_H_30_O_14_	[M+H]+	5.2	Flavones	10
7-hydroxy-3-(4-hydroxyphenyl)-8-((3R,4R,5S,6R)-3,4,5-trihydroxy-6-(hydroxymethyl)tetrahydro-2H-pyran-2-yl)-4H-chromen-4-one	0.73	15.992	433.113	C_21_H_20_O_9_	[M+H]+	5.2	Flavones	8
Kaempferol	0.80	0.000	287.055	C_15_H_10_O_6_	[M+H]+	5.3	Flavonols	11
Apigenin-8-C-glucoside-2′-rhamnoside	0.79	30.009	609.18	C_27_H_30_O_14_	[M+H]+	5.3	Flavones	9
Epicatechin gallate	0.91	0.024	465.104	C_22_H_18_O_10_	[M+Na]	5.5	Flavones	3
Luteolin 4′-O-glucoside	0.97	0.001	449.109	C_21_H_20_O_11_	[M+H]+	5.7	Flavones	8
Petunidin-3-O-B-glucopyranoside	0.89	0.001	479.119	C_22_H_22_O_12_	[M+H]+	5.8	Anthocyanin glycosede	5
Kaempferol	0.80	0.000	287.055	C_15_H_10_O_6_	[M+H]+	6.7	Flavonols	11
Aspergillusenes A	0.71	0.001	235.169	C_15_H_22_O_2_	[M+H]+	8.8	Sesquiterpenoid	11
Luteolin-8-glucoside analog	0.79	15.990	595.167	C_27_H_30_O_15_	[M+H]+	4.9/5.2	Flavones	16

*Momordica charantia* hydroethanolic leaf extracts (MCHLE). Retention Time (RT). Manual Annotation (MA). Molecular formula not assigned (NA). * The more intense fragment ions are described at most.

**Table 2:** Biochemical parameters of *Wistar* rats after treatment of Acute toxicity and Repeated Dose toxicity with *Momordica charantia* hydroethanolic leaf extract (MCHLE).

Biochemical Parameters	Acute Toxicity		Repeated Dose Toxicity							
	Male		Female				Male			
	Control	2000 mg/kg	Control	100 mg/kg	200 mg/kg	400 mg/kg	Control	100 mg/kg	200 mg/kg	400 mg/kg
Gluc (mg/dL)	152 ± 8.91	73 ± 1.1 *	124.34 ± 5.32	122.05 ± 12.32	100.09 ± 15.02 *^,#^	40.94 ± 1.32 *^,#^	121.73 ± 12.09	100.08 ± 2.13 ^#^	85.43 ± 1.32 *^,#^	60.02 ± 1.35 *^,#^
Trig (mg/dL)	78 ± 1.65	35.1 ± 3.35	21.80 ± 1.80	23.99 ± 2.77	24.23 ± 1.09	24.98 ± 1.23	40.21 ± 1.12	41.12 ± 1.34	45.01 ± 3.76	43.23 ± 1.63
Chol (mg/dL)	58.2 ± 5.01	29 ± 0.97 *	63.63 ± 4.32	61.63 ± 4.32	60.01 ± 1.22	32.76 ± 2.98 *	50.21 ± 2.42	50.43 ± 1.02	53.02 ± 1.95	24.93 ± 1.88 *
ALT (U/L)	95 ± 4.44	93 ± 1.60	86.34 ± 5.64	88.32 ± 15.06	86.92 ± 3.43	84.65 ± 9.08	85.23 ± 1.54	84.65 ± 1.01	84.52 ± 2.98	83.12 ± 5.01
AST (U/L)	234 ± 21.3	215 ± 18.5	215.98 ± 1.39	214.65 ± 9.38	210.23 ± 5.39	212 ± 2.98	200.23 ± 13.38	201.62 ± 5.35	200.98 ± 1.54	203.76 ± 3.79
γ-GT (U/L)	14 ± 0.23	13.3 ± 0.01	11.98 ± 0.54	10.87 ± 0.05	10.87 ± 0.50	11.77 ± 0.02	9.89 ± 0.32	9.88 ± 1.51	9.32 ± 0.02	9.32 ± 0.32
TB (mg/dL)	0.33 ± 0.01	0.20 ± 0.01	0.46 ± 0.02	0.25 ± 0.05	0.34 ± 0.01	0.55 ± 0.25	0.35 ± 0.15	0.35 ± 0.25	0.30 ± 0.02	0.32 ± 0.05
DB (mg/dL)	0.103 ± 0.04	0.104 ± 0.09	1.34 ± 0.01	1.32 ± 0.01	1.38 ± 0.01	1.18 ± 0.01	1.43 ± 0.05	1.45 ± 0.01	1.35 ± 0.52	1.34 ± 0.08
IB (mg/dL)	0.25 ± 0.01	0.21 ± 0.10	0.20 ± 0.001	0.17 ± 0.001	0.14 ± 0.005	0.17 ± 0.001	0.18 ± 0.002	0.10 ± 0.001	0.05 ± 0.001	0.02 ± 0.001
Urea (mg/dL)	50.5 ± 1.7	43.3 ± 1.04	34.23 ± 1.21	33.84 ± 2.61	31.77 ± 1.81	32.54 ± 2.09	32.23 ± 0.01	32.87 ± 1.61	31.77 ± 1.81	31.54 ± 1.02
Uric acid (mg/dL)	1.14 ± 0.45	1.01 ± 0.01	1.10 ± 0.01	1.15 ± 0.04	1.15 ± 0.05	1.01 ± 0.232	1.6 ± 0.05	1.15 ± 0.06	1.23 ± 0.41	1.48 ± 0.99

Data are expressed as the mean ± standard deviation (SD). The control group received the vehicle (distilled water). Statistical comparisons among groups were performed using one-way ANOVA followed by Tukey’s post hoc test. Sample sizes were n = 3 for the acute oral toxicity assay and n = 5 for the repeated-dose toxicity assay. The biochemical parameters evaluated included glucose (Gluc), triglycerides (Trig), cholesterol (Chol), alanine aminotransferase (ALT), aspartate aminotransferase (AST), gamma-glutamyl transferase (γ-GT), total bilirubin (TB), direct bilirubin (DB), and indirect bilirubin (IB). * *p* < 0.05 indicates a statistically significant difference compared with the control group within the same experiment. ^#^ *p* < 0.05 indicates a statistically significant difference among treatment groups (100, 200, and 400 mg/kg).

**Table 3:** Effects of *Momordica charantia* hydroethanolic leaf extract (MCHLE) on relative organ weight in experiments of Acute oral toxicity and Repeated Dose oral toxicity in Wistar rats.

Organ (g)	Acute Toxicity		Repeated Dose Toxicity							
	Male		Female				Male			
	Control	2000 mg/kg	Control	100 mg/kg	200 mg/kg	400 mg/kg	Control	100 mg/kg	200 mg/kg	400 mg/kg
Kidney (R)	0.30 ± 0.01	0.3 ± 0.03	0.45 ± 0.04	0.54 ± 0.12	0.52 ± 0.14	0.55 ± 0.90	0.38 ± 0.17	0.45 ± 0.15	0.39 ± 0.16	0.40 ± 0.01
Kidney (L)	0.31 ± 0.03	0.31 ± 0.1	0.36 ± 0.09	0.40 ± 0.10	0.65 ± 0.14	0.65 ± 0.54	0.35 ± 0.08	0.44 ± 0.05	0.38 ± 0.10	0.30 ± 0.00
Liver	4.21 ± 0.5	3.96 ± 0.03	4.54 ± 0.08	4.98 ± 0.12	4.35 ± 0.15	4.99 ± 0.12	4.54 ± 0.05	4.00 ± 0.09	4.00 ± 0.38	4.40 ± 0.02
Spleen	0.14 ± 0.01	0.13 ± 0.01	0.34 ± 0.04	0.54 ± 0.03	0.43 ± 0.03	0.47 ± 0.04	0.26 ± 0.05	0.28 ± 0.01	0.25 ± 0.08	0.20 ± 0.09
Heart	0.21 ± 0.01	0.20 ± 0.02	0.21 ± 0.02	0.34 ± 0.05	0.45 ± 0.09	0.25 ± 0.04	0.26 ± 0.17	0.24 ± 0.07	0.26 ± 0.08	0.30 ± 0.05
Lung	0.42 ± 0.01	0.5 ± 0.01	0.41 ± 0.04	0.48 ± 0.05	0.43 ± 0.04	0.39 ± 0.03	0.42 ± 0.03	0.48 ± 0.03	0.40 ± 0.04	0.60 ± 0.05
Stomach	0.45 ± 0.14	0.6 ± 0.21	0.54 ± 0.02	0.66 ± 0.08	0.55 ± 0.05	0.52 ± 0.03	0.48 ± 0.05	0.42 ± 0.01	0.39 ± 0.02	0.40 ± 0.03

Data are presented as the mean ± standard deviation (SD). Sample sizes were n = 3 for the acute oral toxicity assay and n = 5 for the repeated-dose toxicity assay. The control group received the vehicle (distilled water). Statistical comparisons among groups were performed using one-way ANOVA followed by Tukey’s post hoc test, revealing no significant differences. R = right; L = left.

**Table 4:** Effects of *Momordica charantia* hydroethanolic leaf extract (MCHLE) on antioxidative parameters and lipid peroxidation (TBARS) in experiments of Acute oral toxicity and Repeated Dose oral toxicity in liver tissue of *Wistar* rats.

Oxidative Stress Parameters	Acute Toxicity		Repeated Dose Toxicity							
	Male		Female				Male			
	Control	2000 mg/kg	Control	100 mg/kg	200 mg/kg	400 mg/kg	Control	100 mg/kg	200 mg/kg	400 mg/kg
MDA (μmol/L)	78.14 ± 1.12	43.44 ± 1.55	80.23 ± 3.43	80.43 ± 1.57	40.84 ± 1.23 *	30.43 ± 1.43 *	74.16 ± 1.02	73.51 ± 1.78 *	42.10 ± 1.24 *	29.19 ± 1.36 *
GSH (μmol/L)	5.48 ± 0.05	4.93 ± 0.06	5.65 ± 1.32	5.73 ± 0.04	4.98 ± 0.01	5.46 ± 0.03	5.51 ± 0.01	5.54 ± 0.07	5.93 ± 0.08	2469 ± 0.08
SOD (IU/mg protein)	U.R.	U.R.	68.34 ± 1.43	65.56 ± 0.43	65.23 ± 1.43	88.35 ± 1.23 *	68.30 ± 0.04	69.34 ± 0.21	66.32 ± 1.39	85.50 ± 1.58 *
GPx (IU/mg protein)	U.R.	U.R.	5.74 ± 0.05	7.58 ± 0.08 *	8.43 ± 0.01 *	10.98 ± 0.03 *	5.91 ± 0.06	5.19 ± 0.07	7.56 ± 0.02 *	11.01 ± 0.04 *

Values are expressed as the mean ± standard deviation (SD). Sample sizes were n = 3 for the acute oral toxicity test and n = 5 for the repeated-dose oral toxicity test. Group comparisons were conducted using one-way ANOVA followed by Tukey’s post hoc test. The biochemical markers evaluated included malondialdehyde (MDA), reduced glutathione (GSH), superoxide dismutase (SOD), and glutathione peroxidase (GPX). * *p* < 0.05 indicates a statistically significant difference compared with the control group within the same experiment. U = unrealized.

## Data Availability

No new data were created or analyzed in this study. Data sharing is not applicable to this article.

## Supplementary Materials

The following supporting information can be downloaded at: https://www.mdpi.com/article/10.3390/molecules30224335/s1. Figure S1. Comparison between library GNPS (bottom) and query spectra phytocomponents identified in MCHLE (top). The structure of the phytocomponent putatively identified is represented. and Table S1. List of genes related to inflammation used in RT-PCR.
