# Supplementary material for: Momordica charantia L. (Cucurbitaceae) Leaf Extract from Phytochemical Characterization and Toxicity Evaluation to Modulation of Pro-Inflammatory Cytokines and MAPK/NFκB Pathways
Source: Molecules. 2025 Nov 7;30(22):4335. doi: 10.3390/molecules30224335 (PMC12655299; doi:10.3390/molecules30224335)
Supplement: Supplementary file 1 [file molecules-30-04335-s001.zip › molecules-3928752-supplementary.pdf]

Supplementary material

Momordica charantia L. (Cucurbitaceae) Leaf Extract from Phytochemical Characterization and Toxicity Evaluation to Modulation of Pro-Inflammatory Cytokines and MAPK/NFκB Pathways

Figure S1. COMPARISON BETWEEN LIBRARY GNPS (BOTTOM) AND QUERY SPECTRA PHYTOCOMPONENTS IDENTIFIED IN MCHLE (TOP). THE STRUCTURE OF THE PHYTOCOMPONENT PUTATIVELY IDENTIFIED IS REPRESENTED.

Trigonelline

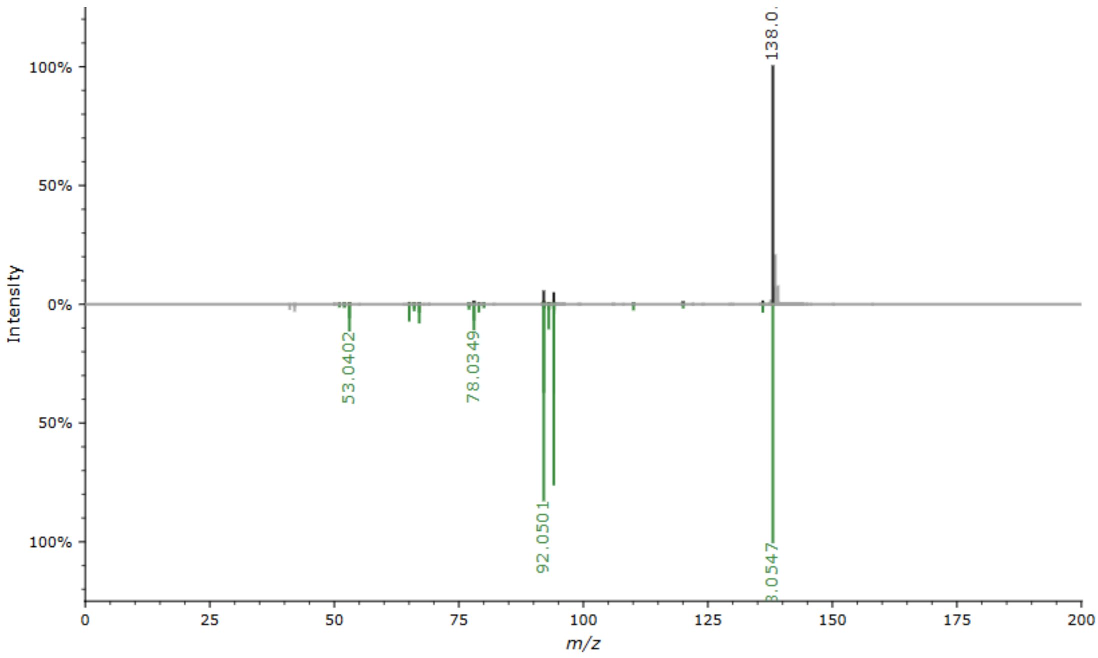

Polysaccharide

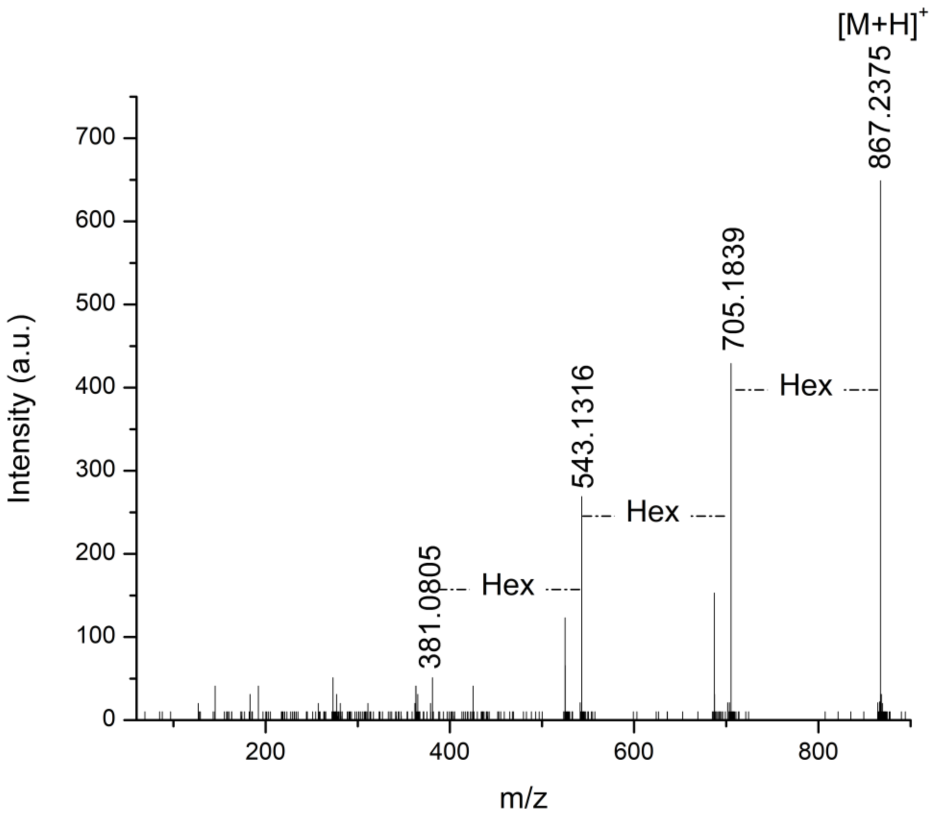

DL-Octopamine

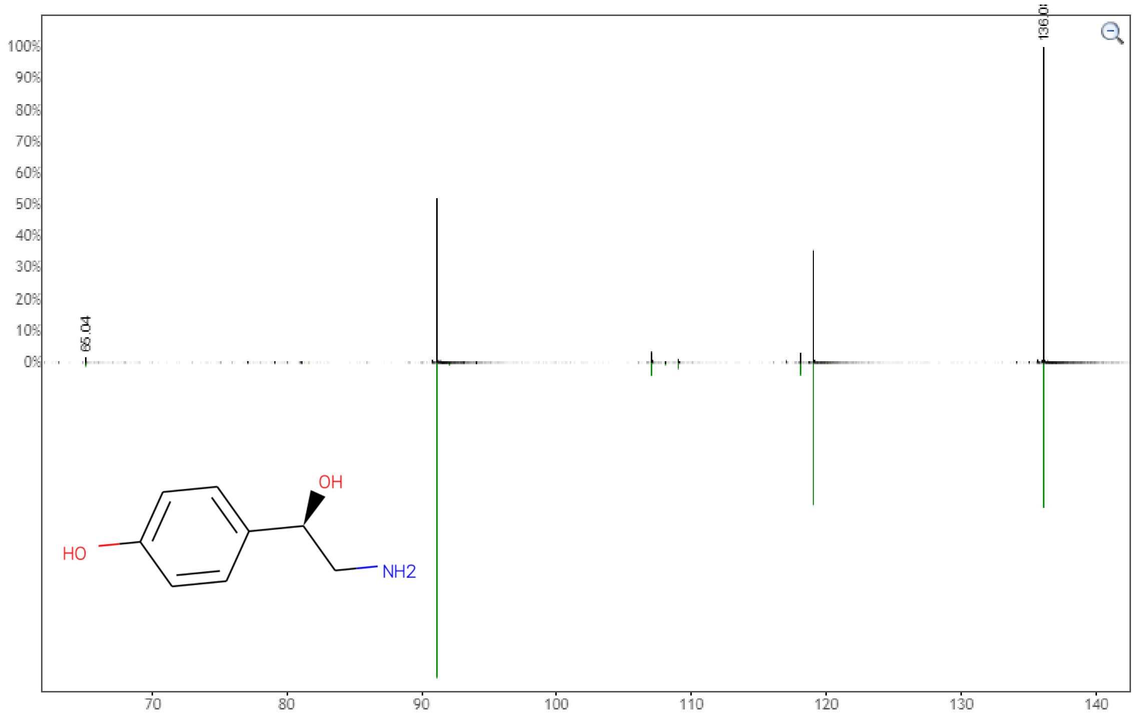

L-Tyrosine

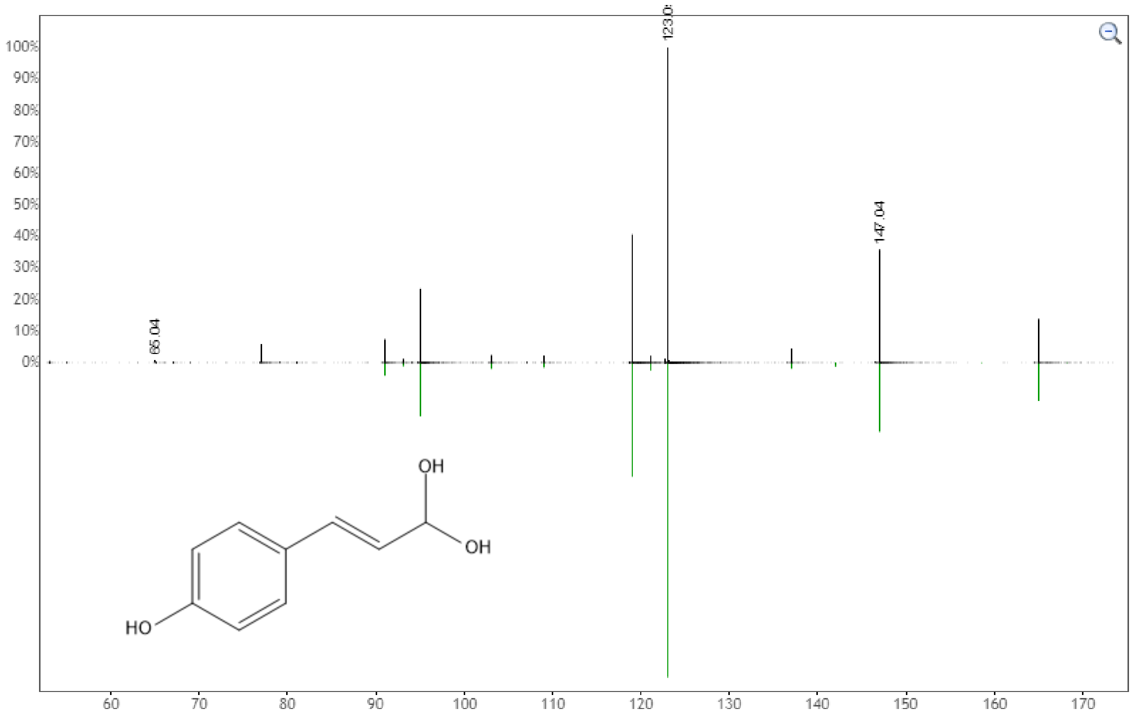

L-(+)-norleucine

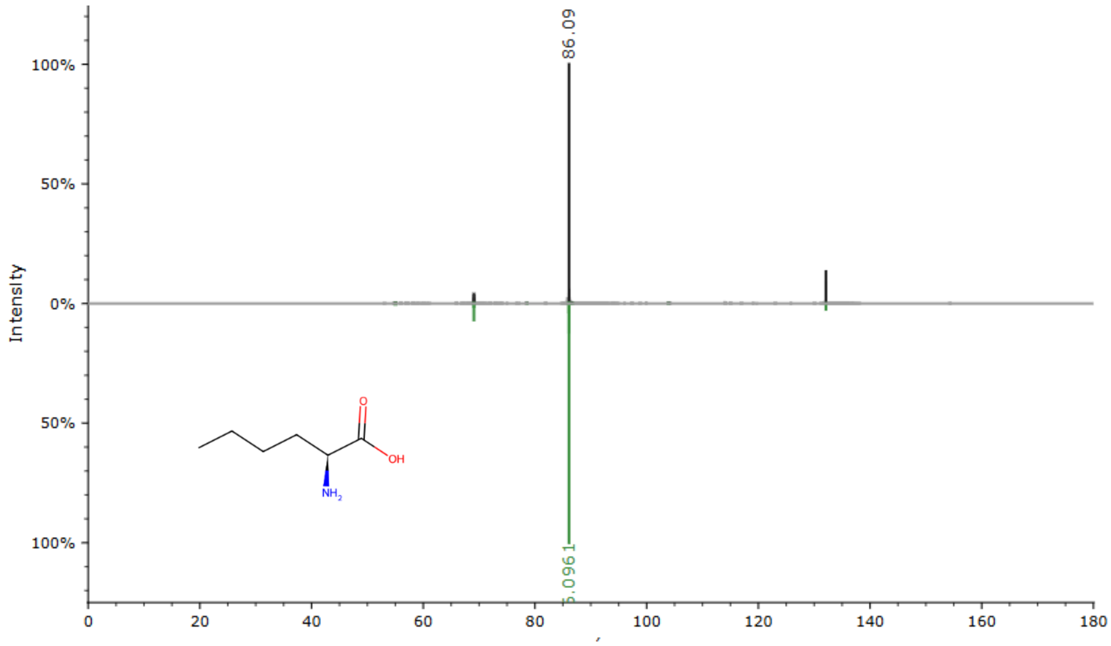

Ciclo(His-Pro)

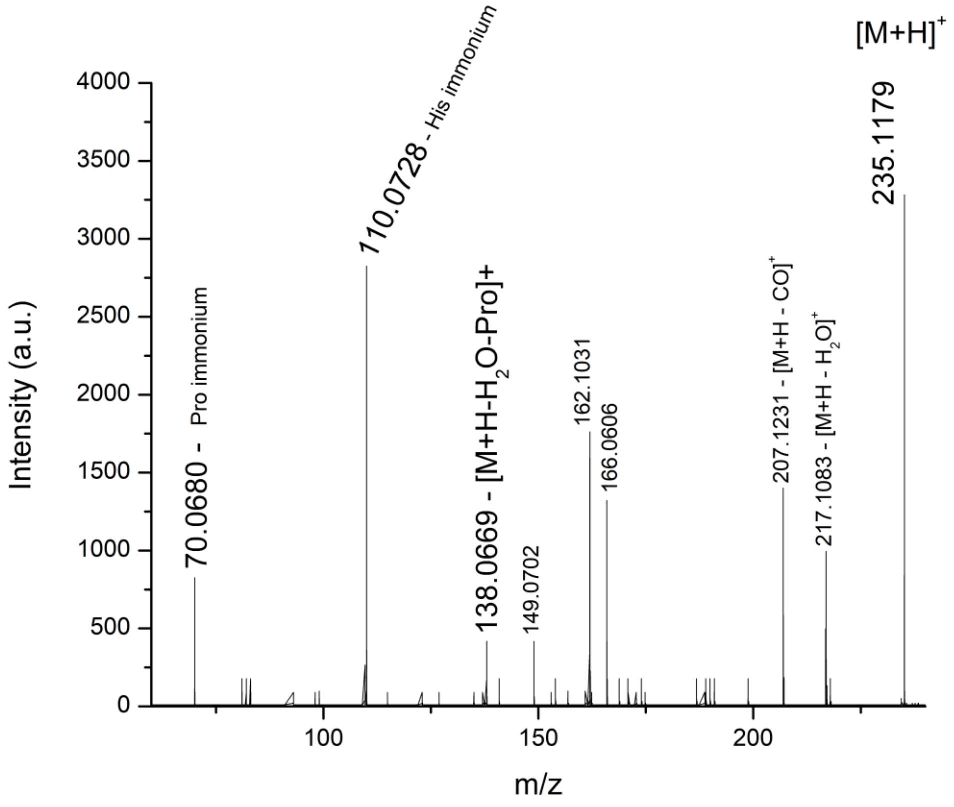

N-fructosyl tyrosine

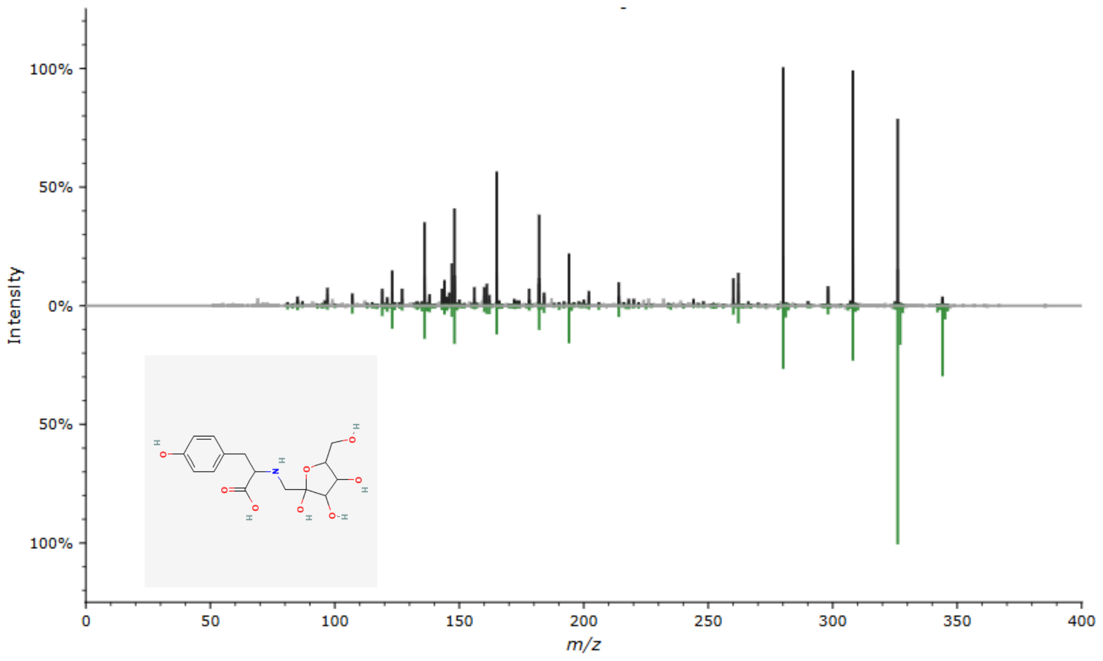

Fru-Gly-Leu/Ile

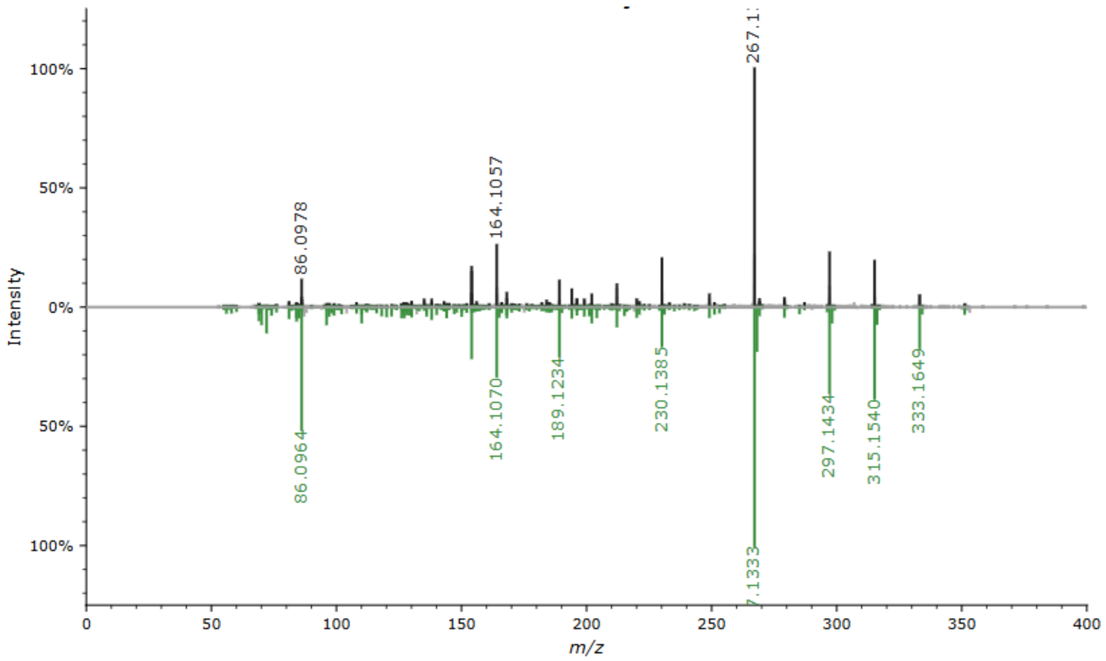

Deoxycarnitine

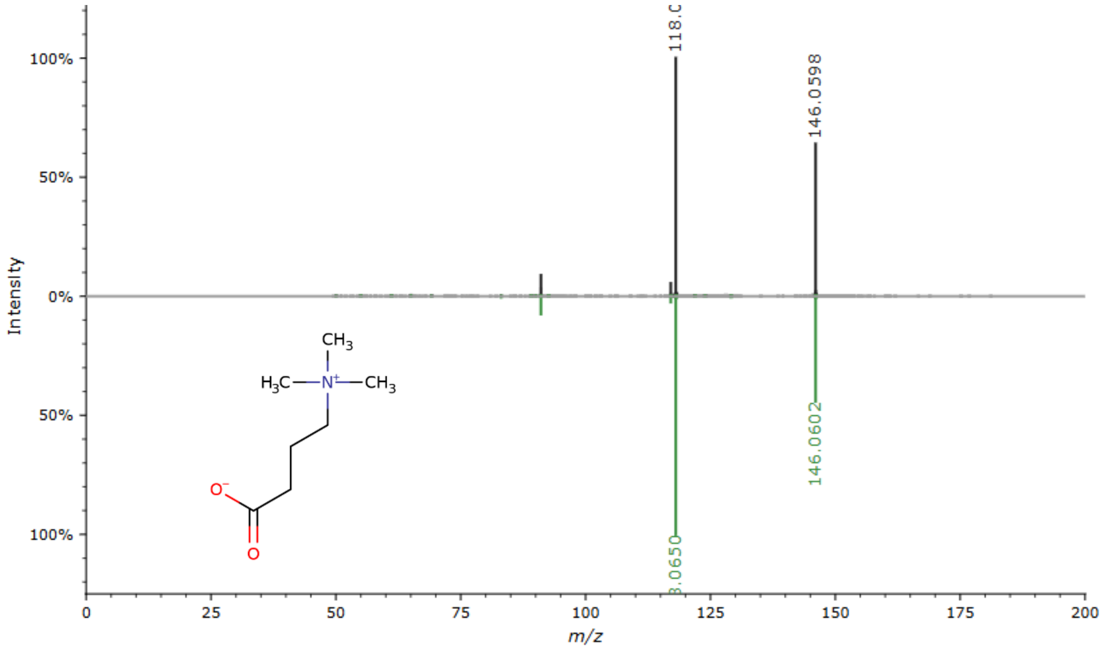

Phenylalanine

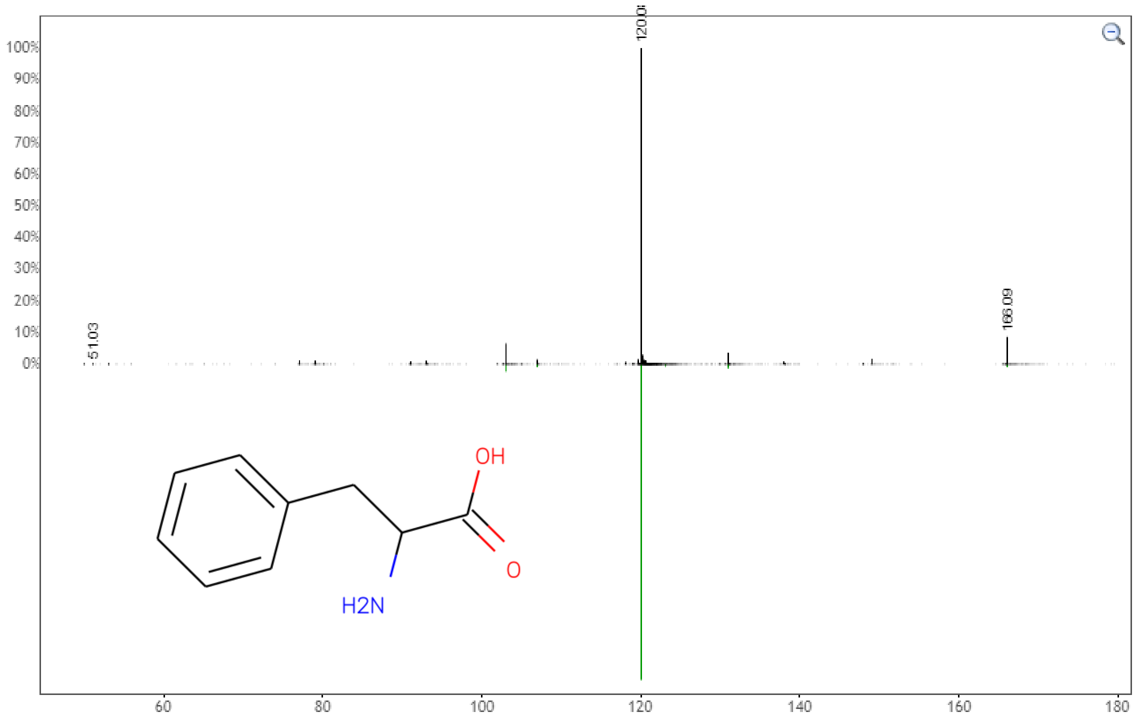

L-Tryptophan

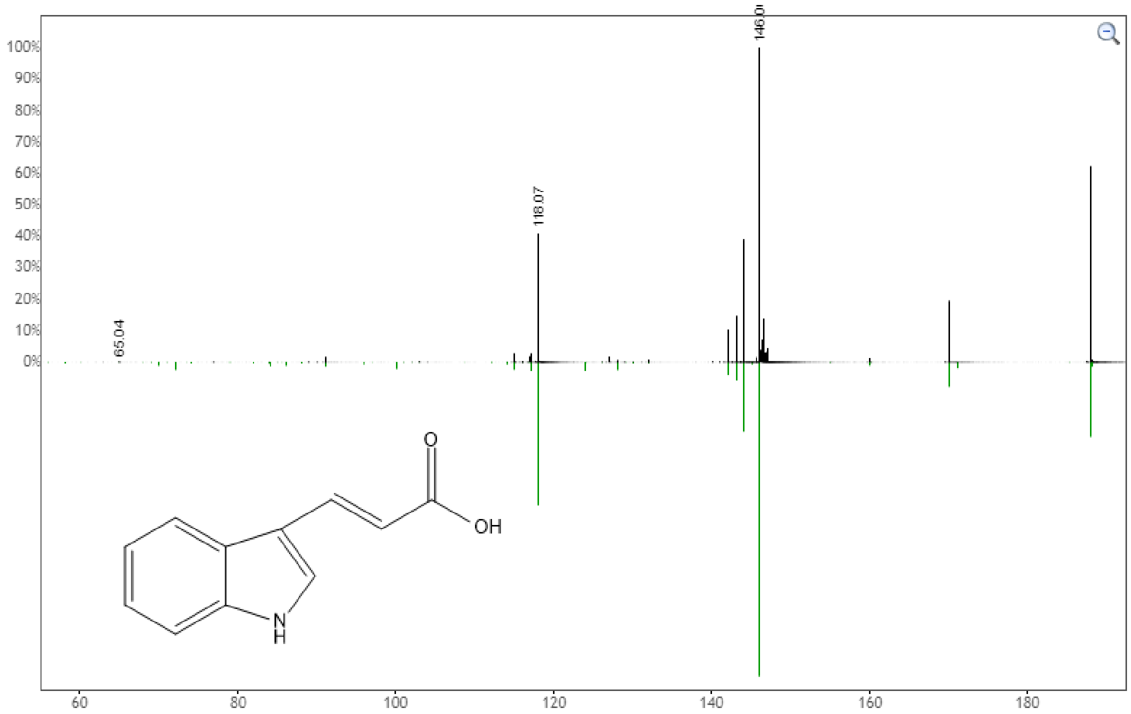

Adenosine, 5_-S-methyl-5_-thio-

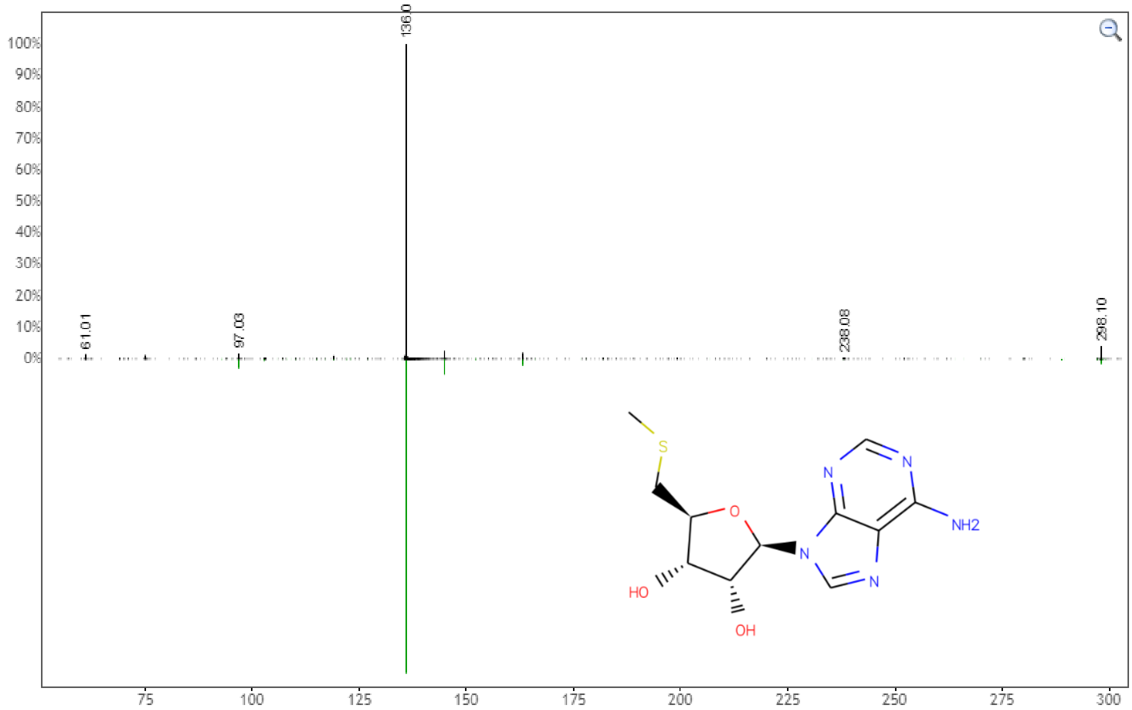

Ferulate

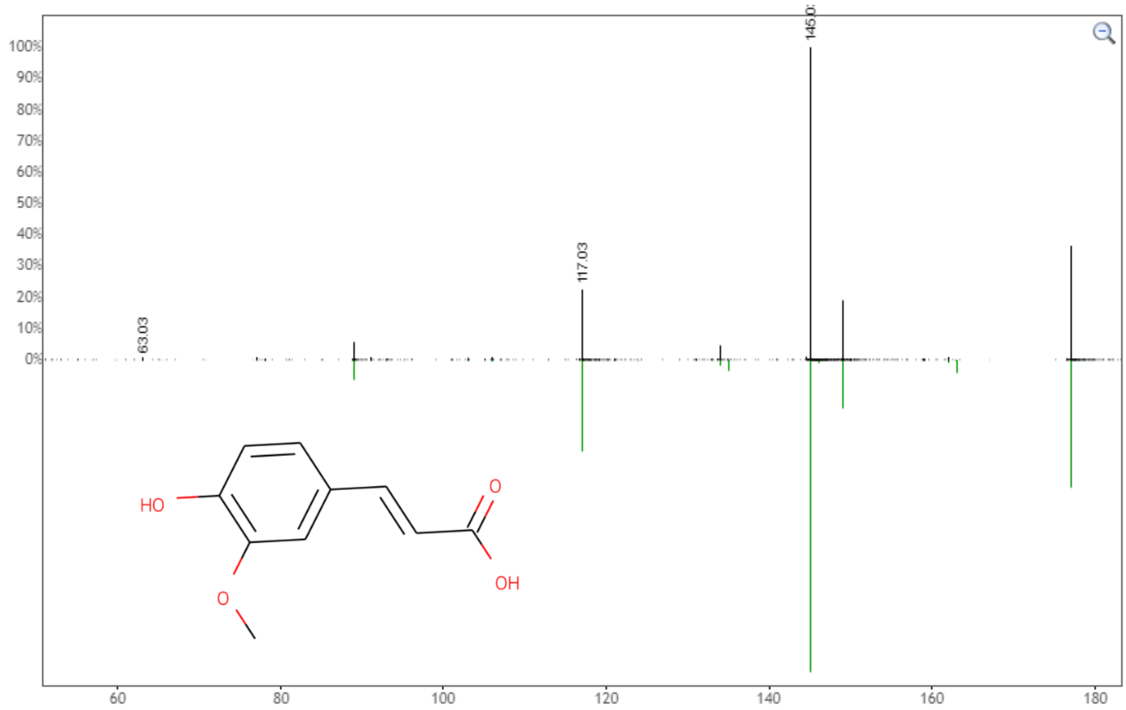

O,C-rhamnosyl-glucosyl-luteolin

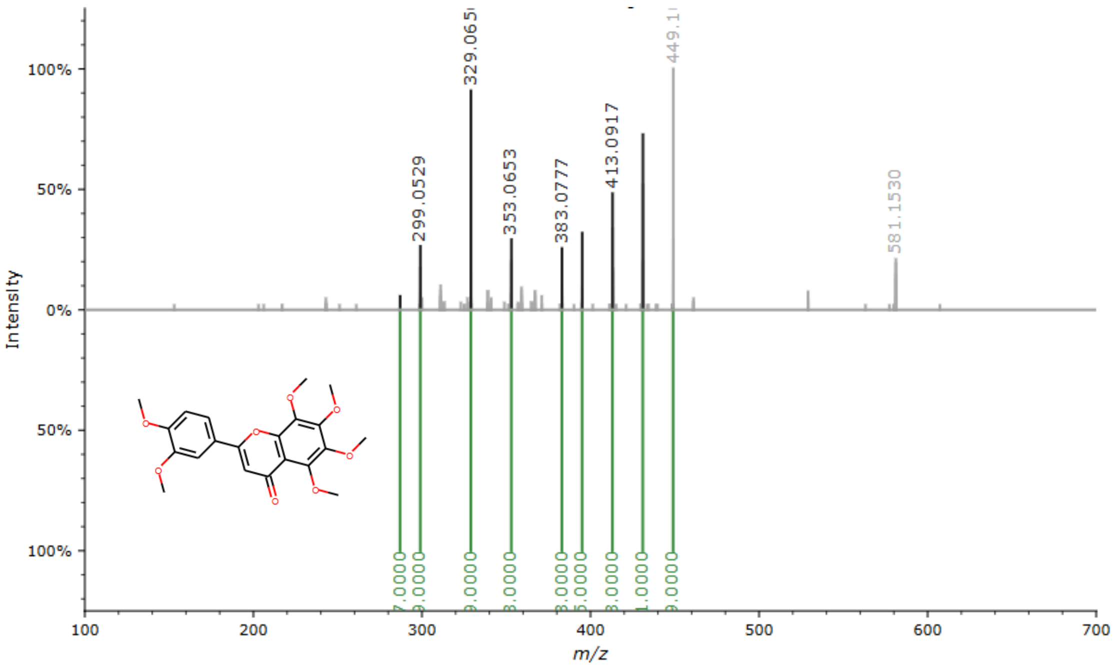

Vitexin-2"-rhamnoside (m/z 659.128)

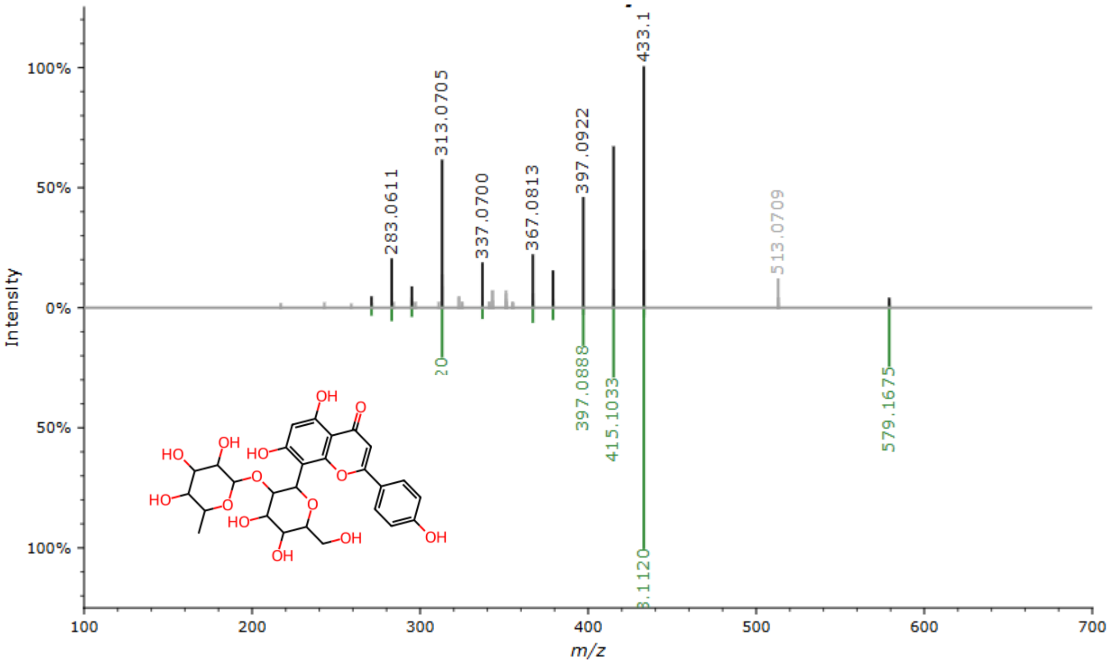

RIBOFLAVIN

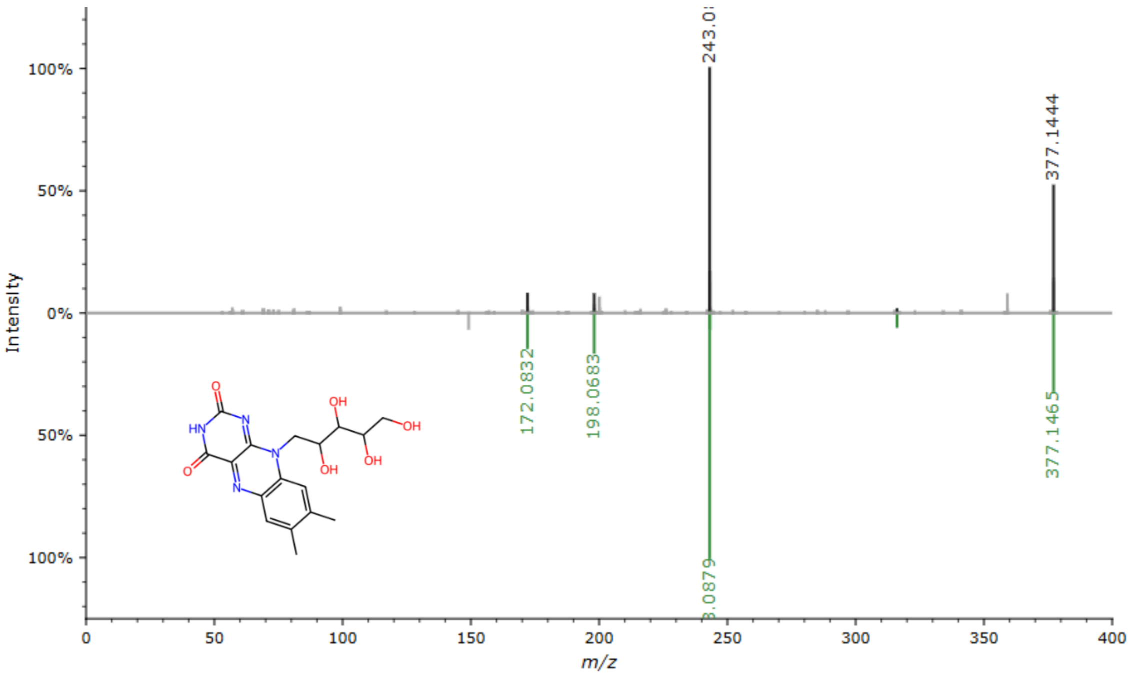

Apigenin-8-C-glucoside-2'-rhamnoside (m/z 645.112)

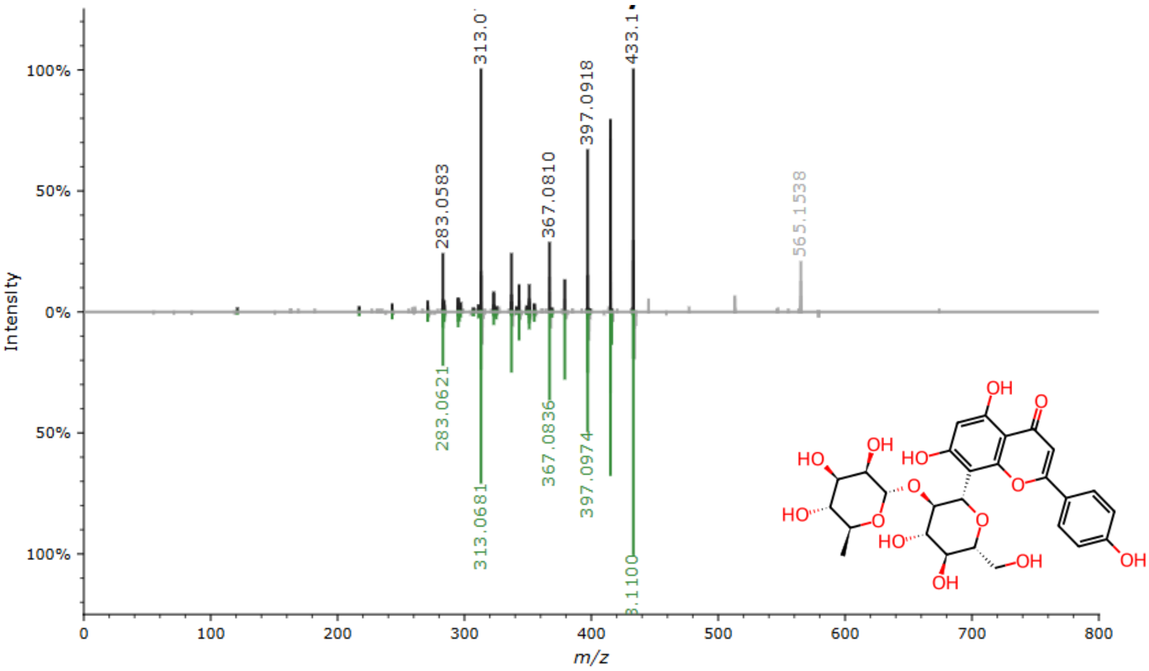

C-Hexosyl-luteolin O-rhamnoside-O-hexoside

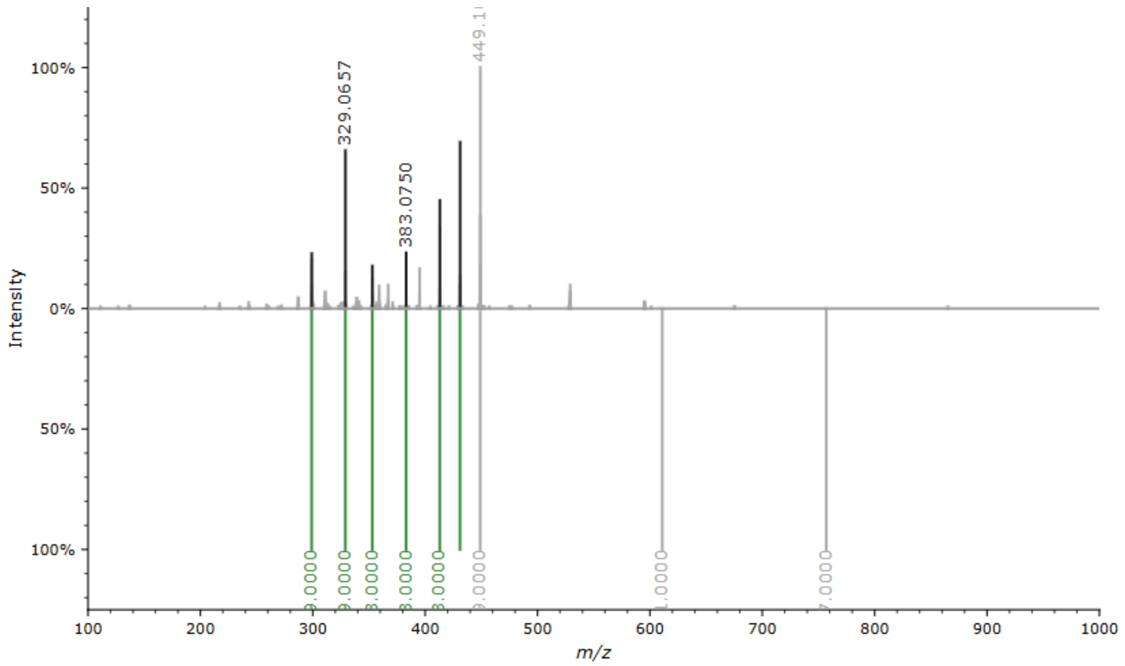

Mirificin

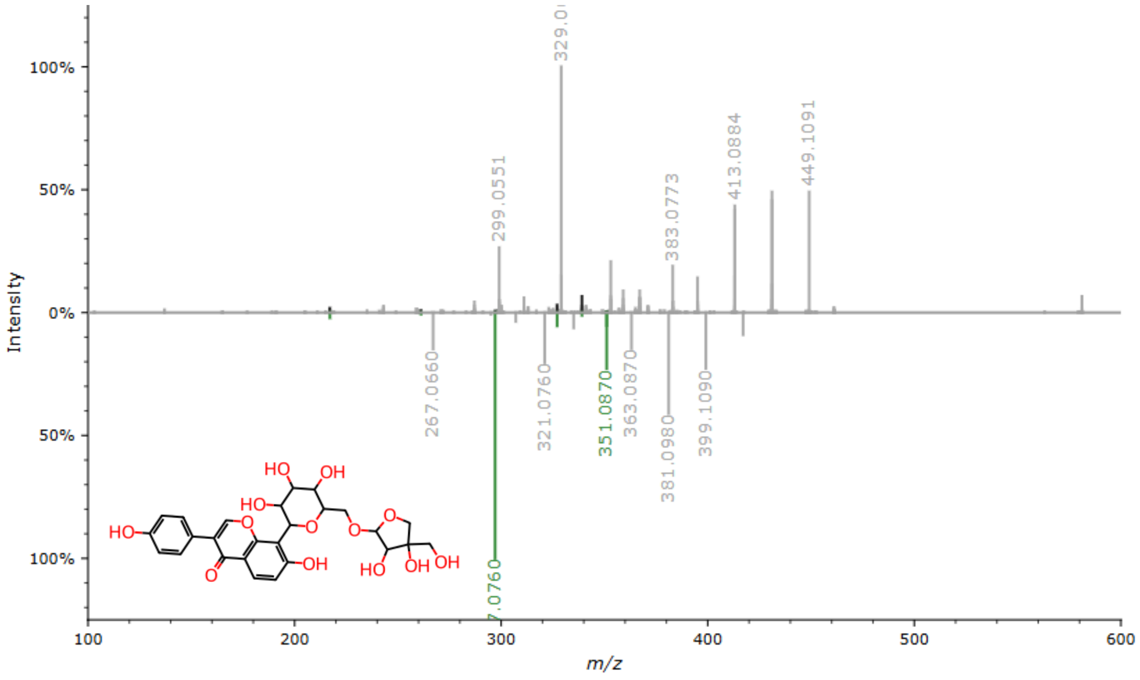

7-O-beta-glucopyranosyl-4'-hydroxy-5-methoxyisoflavone

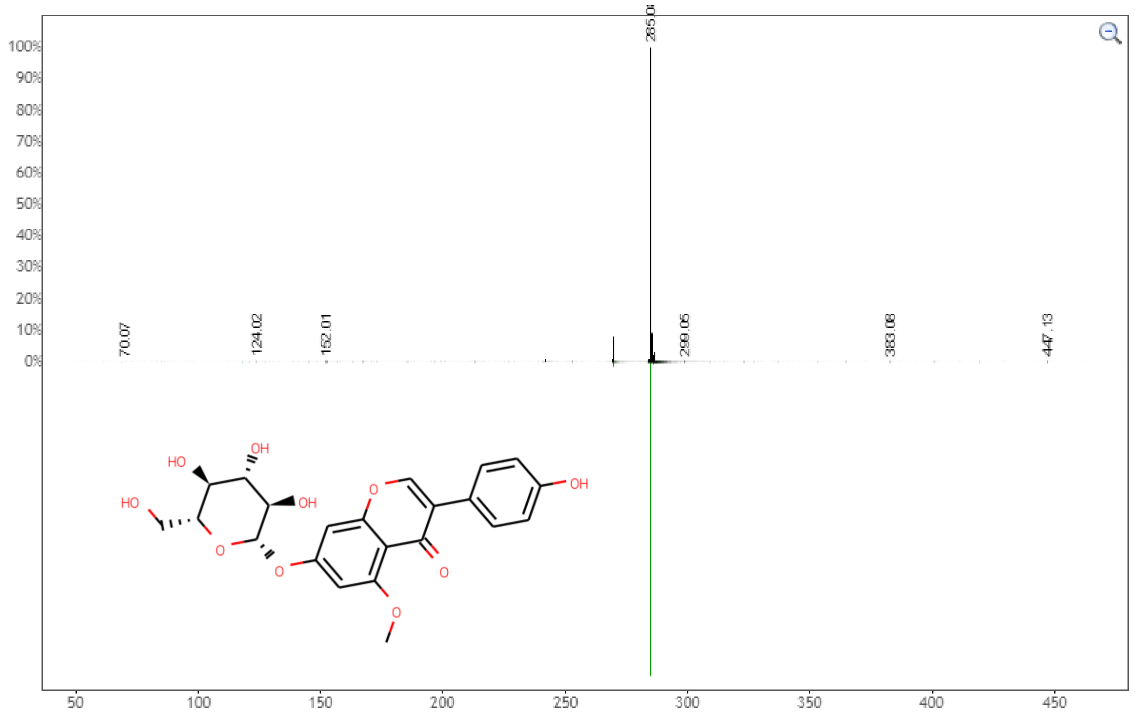

Diprotin A

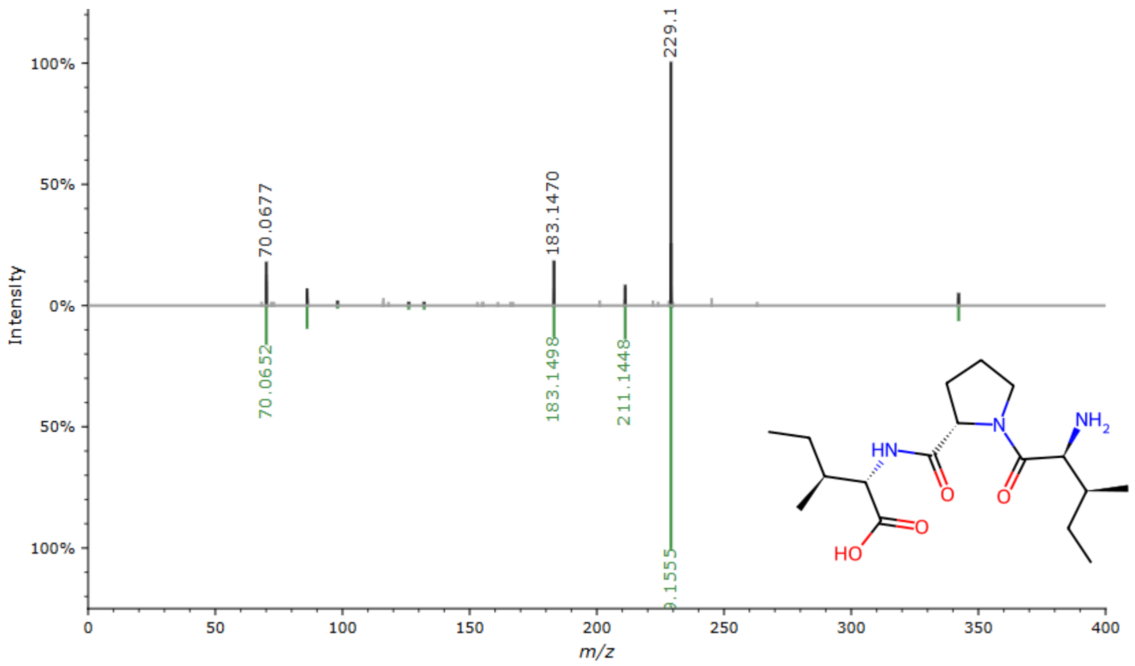

Vitexin-2-O-rhamnoside (579.171)

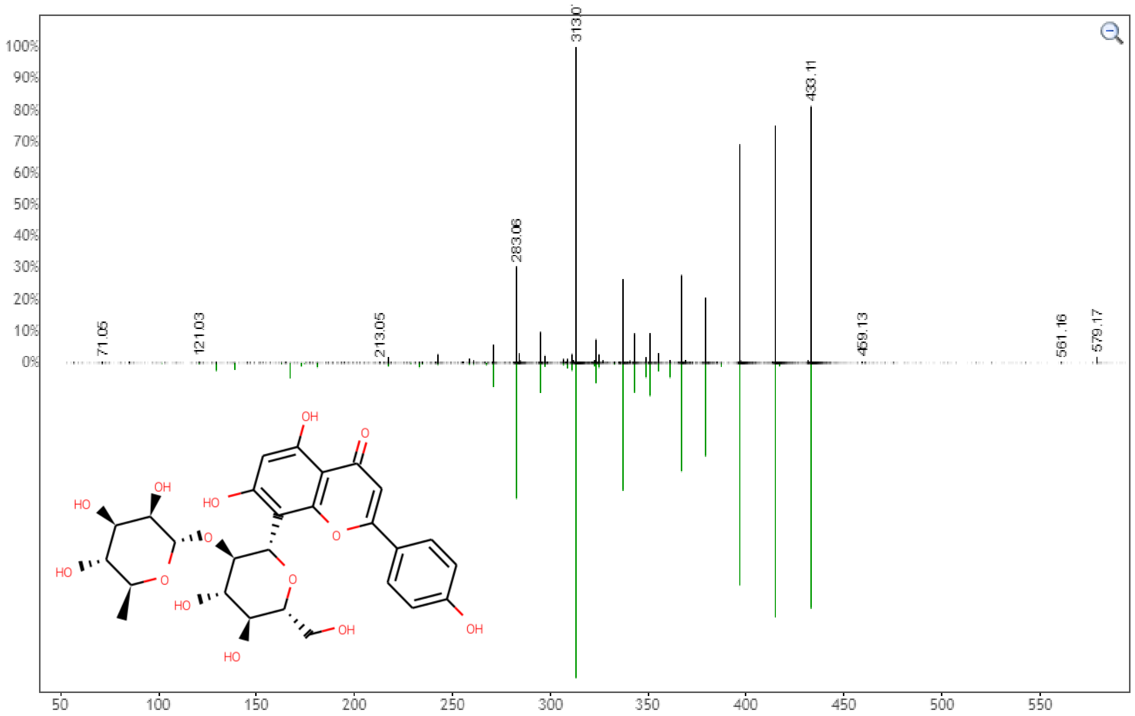

2''-Rhamnosylvitexin

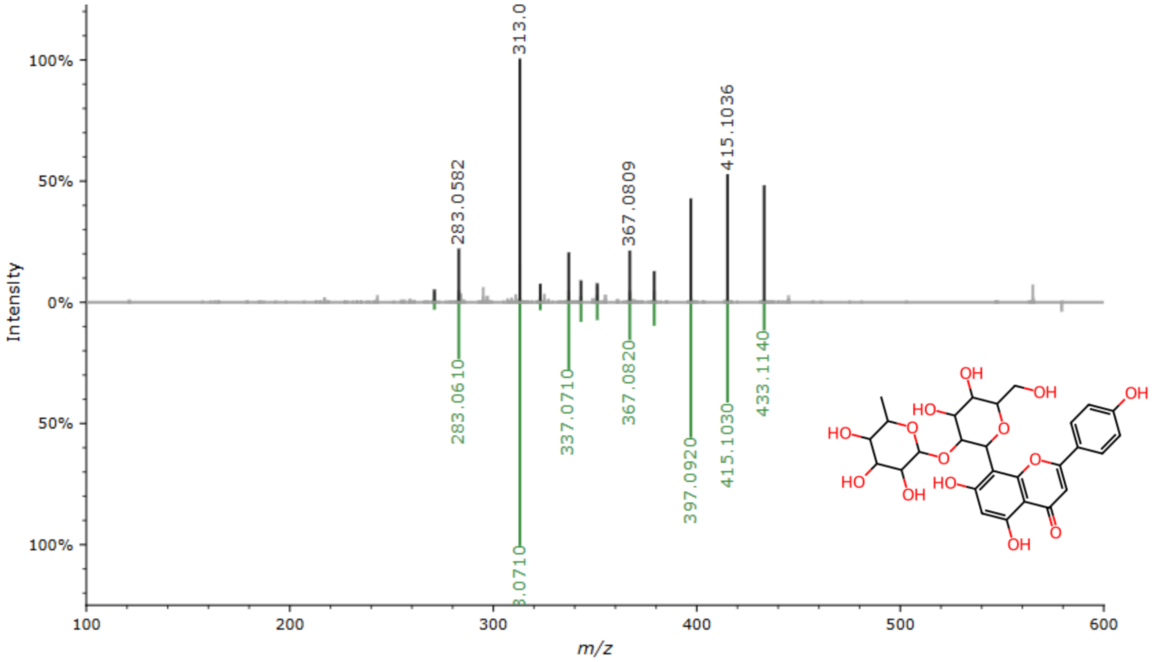

7-hydroxy-3-(4-hydroxyphenyl)-8-((3R,4R,5S,6R)-3,4,5-trihydroxy-6-(hydroxymethyl)tetrahydro-2H-pyran-2-yl)-4H-chromen-4-one

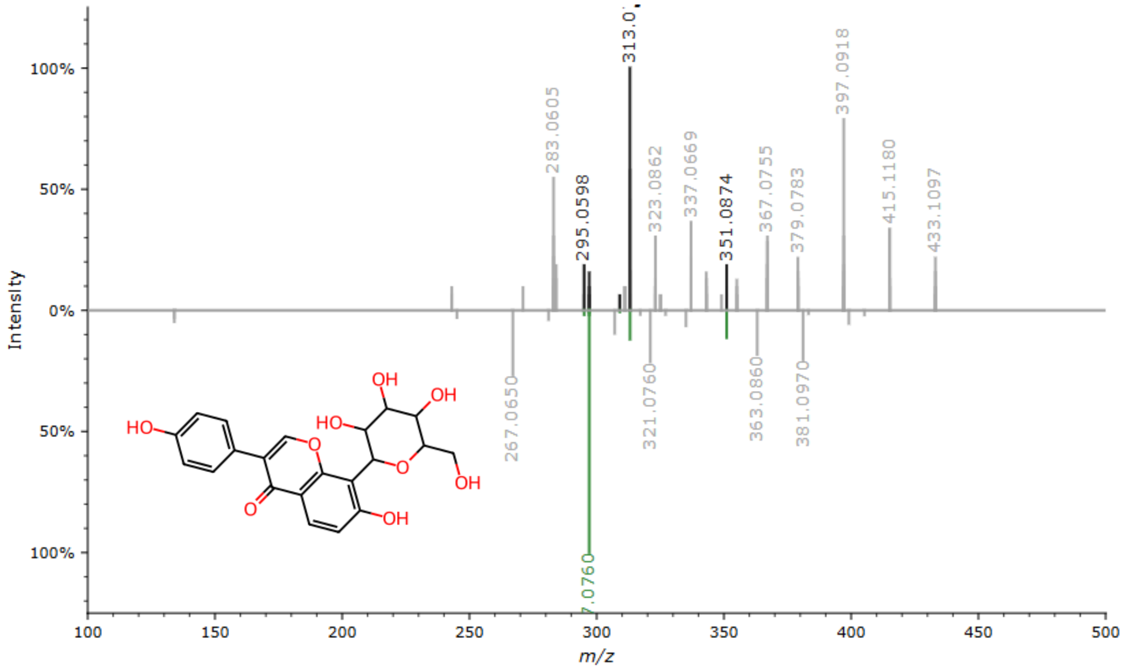

Kaempferol (5.3 min)

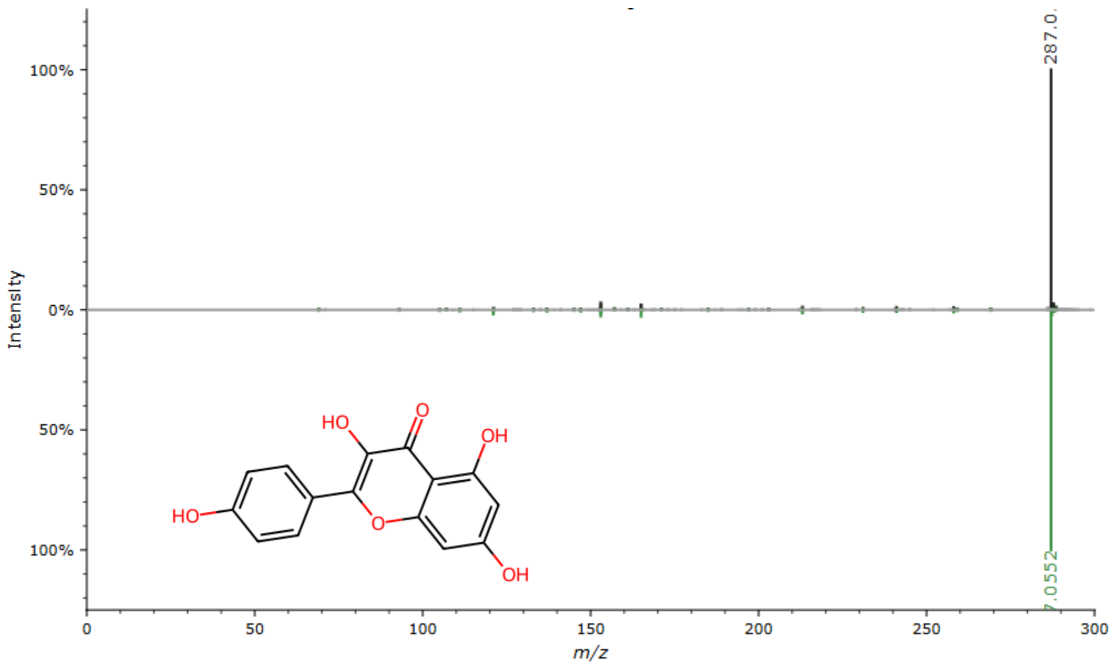

Apigenin-8-C-glucoside-2'-rhamnoside (m/z 609.1800)

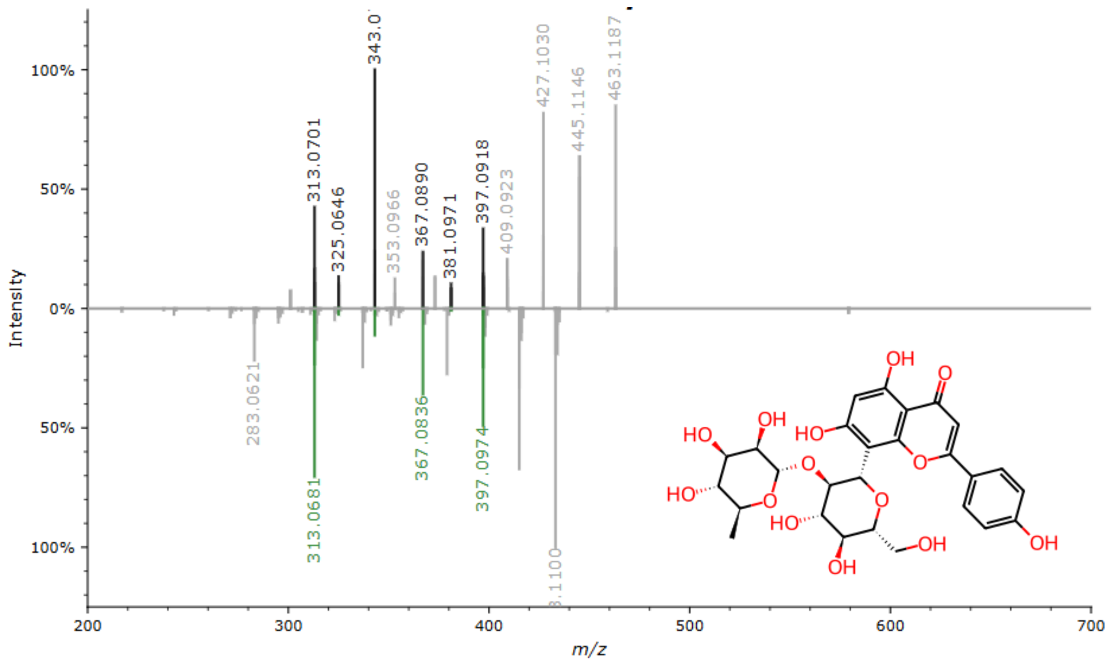

Epicatechin gallate

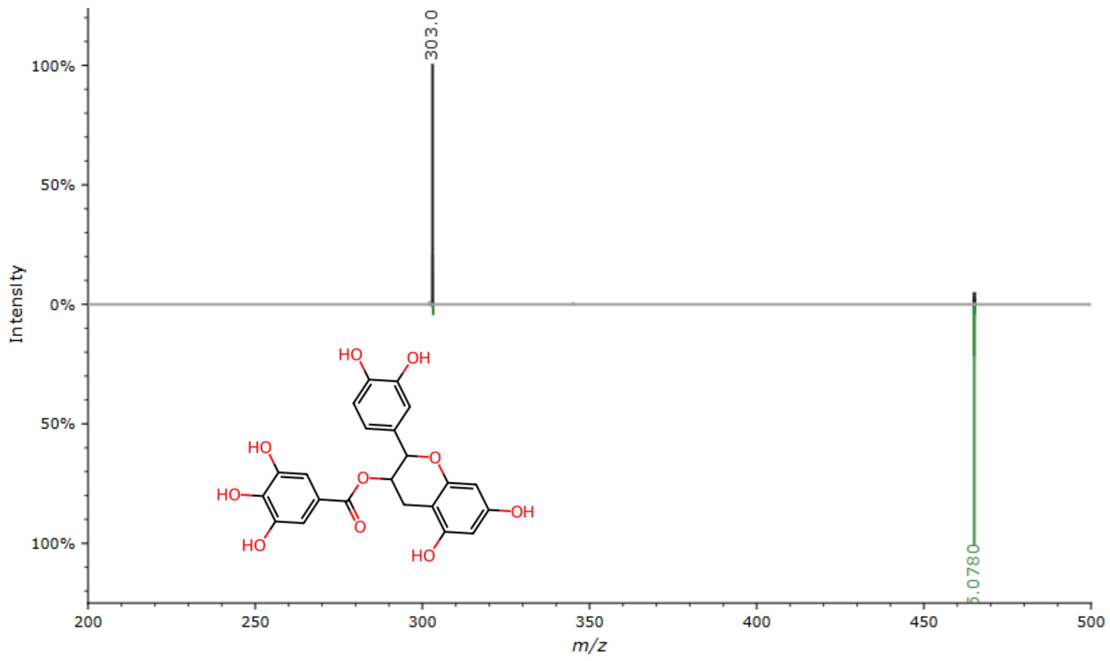

luteolin 4'-O-glucoside

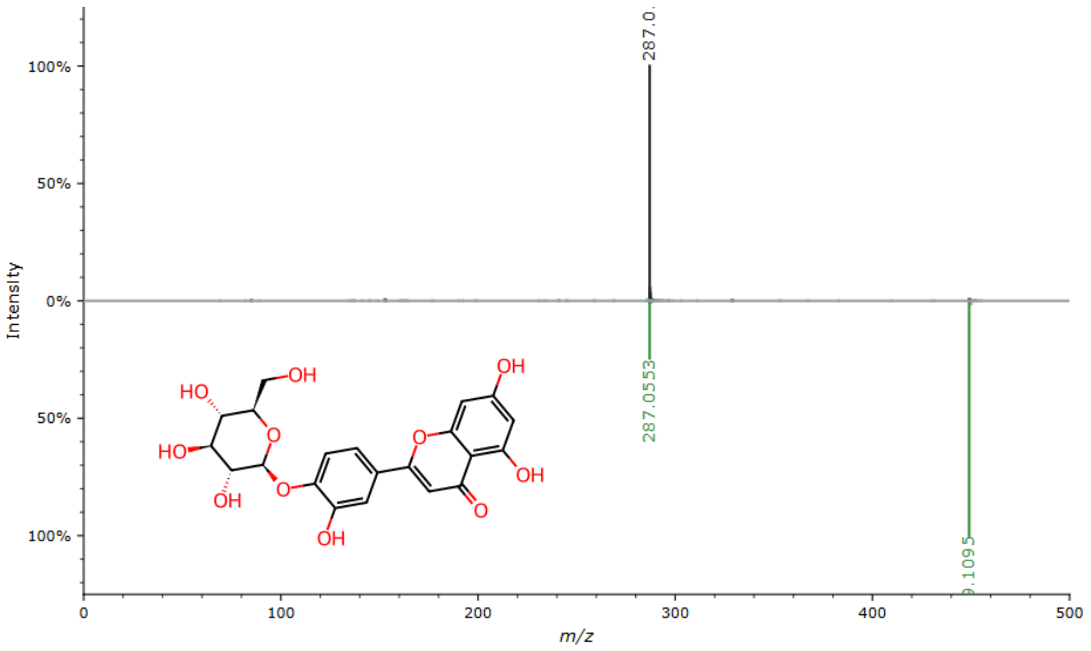

Petunidin-3-O-B-glucopyranoside

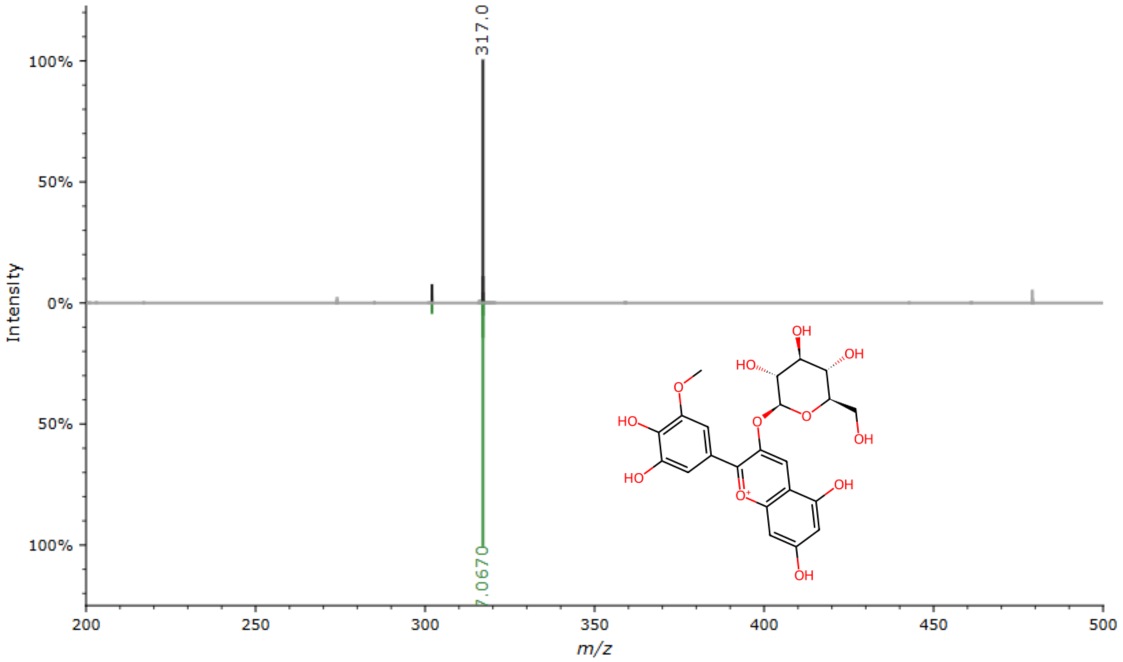

Aspergillusenes A

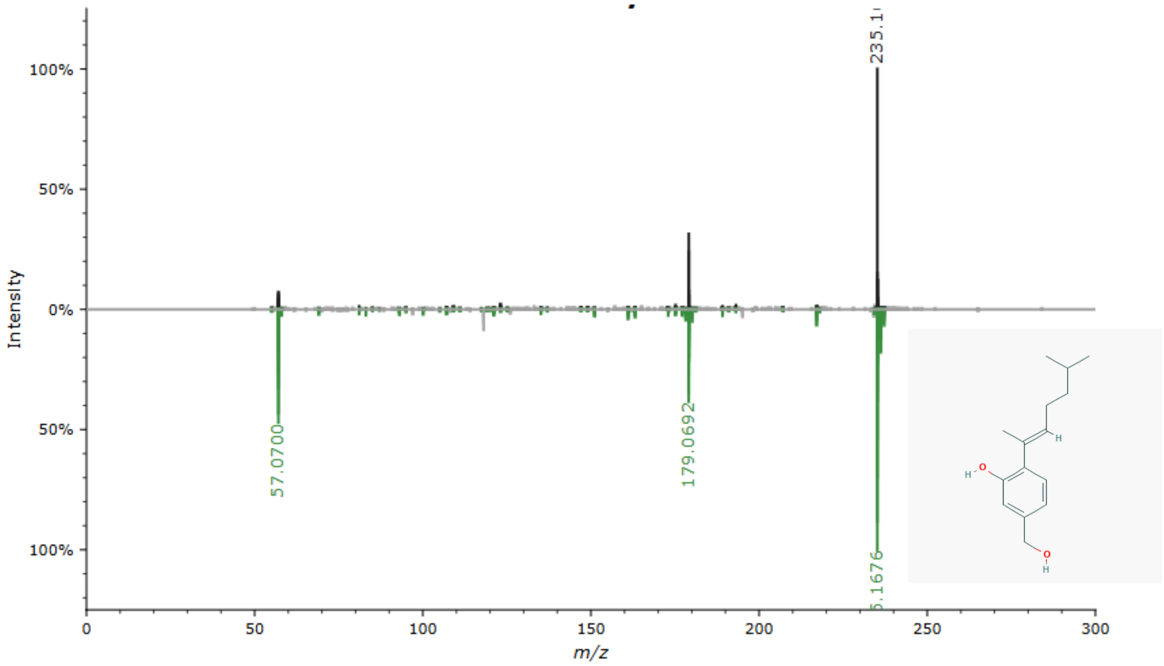

Luteolin-8-glucoside analogue

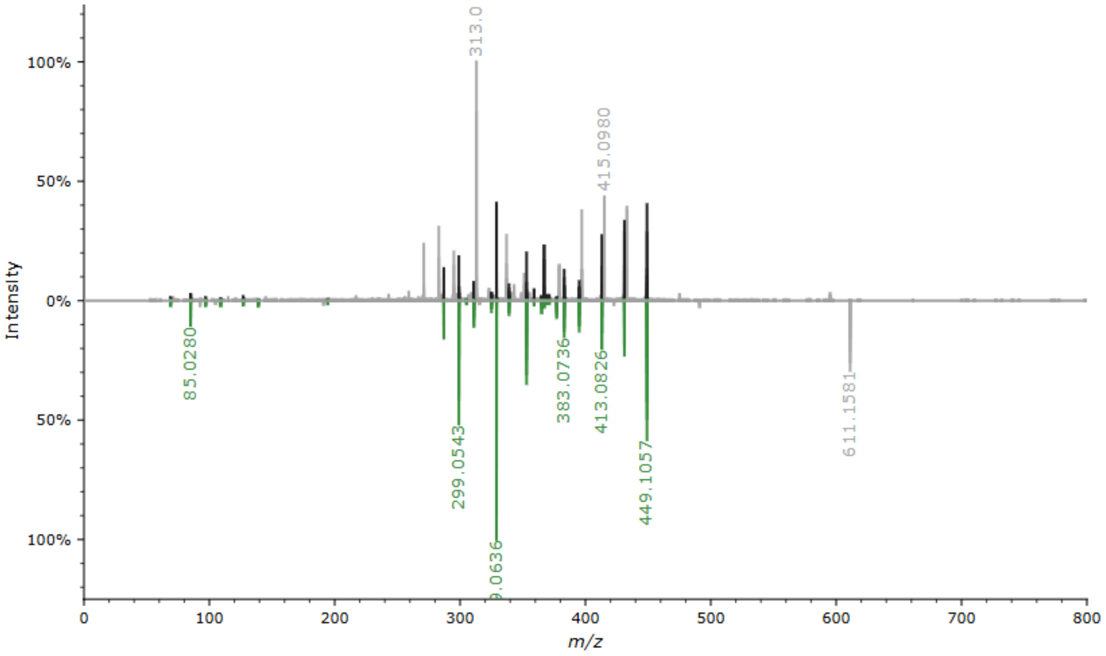

Table S1. LIST OF GENES RELATED TO INFLAMMATION USED IN RT-PCR

Gene	Product	Assay ID
	**Inflammatory Genes**	
**MAPK**	Gene Exp assay (Small) - MAPK1	Mm00442479_m1
**NF-κB**	Gene Exp assay (Small) - NFKB1	Mm00476361_m1
**TNF-α**	Gene Exp assay (Small) - TNF- α	Mm00443258_m1
**IL-1β**	Gene Exp assay (Small) - IL-1β	Mm00434228_m1
**COX2**	Gene Exp assay (Small) - COX2	Mm07306536_g1
	**General Gene**	
**β-actin**	Gene Exp assay (Small) - β-actin	Mm00834862_m1
